# Different explanations for surface and canopy urban heat island effects in relation to background climate

**DOI:** 10.1016/j.isci.2024.108863

**Published:** 2024-01-11

**Authors:** Liu Yang, Qi Li, Qiong Li, Lei Zhao, Zhiwen Luo, Yan Liu

**Affiliations:** 1 State Key Laboratory of Green Building, Department of Architecture, Xi’an University of Architecture and Technology, Xi’an, Shaanxi 710055, P.R. China; 2 State Key Laboratory of Subtropical Building and Urban Science, School of Architecture, South China University of Technology, Guangzhou 510640, P.R. China; 3 Department of Civil and Environmental Engineering, University of Illinois at Urbana-Champaign, Urbana, IL, USA; 4 Welsh School of Architecture, Cardiff University, Cardiff, UK

**Keywords:** Atmospheric science, Climatology, Urban forestry, Urban planning

## Abstract

The background climatic conditions and urban morphology greatly influence urban heat island effects (UHIs), but one-size-fits-all solutions are frequently employed to mitigate UHIs. Here, attribution models for surface UHIs (SUHIs) and canopy UHIs (CUHIs) were developed to describe UHI formation. The contribution of factors to SUHIs and CUHIs shows similar dependencies on background climate and urban morphology. Furthermore, the factors that mainly contributed to CUHIs were more complex, and anthropogenic heat was the more critical factor. Influence from urban morphology also highlights that there is no one-size-fit-all solution for heat mitigation at the neighborhood. In particular, maintaining a low building density should be prioritized, especially mitigating CUHIs. Moreover, it is more effective to prioritize urban irrigation maintenance over increasing green cover in arid regions but the opposite in humid regions. The work can provide scientific evidence to support developing general and regional guidelines for urban heat mitigation.

## Highlights

• Attribution models for SUHIs and CUHIs were developed
• SUHIs and CUHIs show similar dependencies on background climate and urban morphology
• Prioritize maintaining low building density, especially for mitigating CUHIs
• Urban irrigation is more effective than urban greening in arid regions

## Graphical abstract

**Figure:**
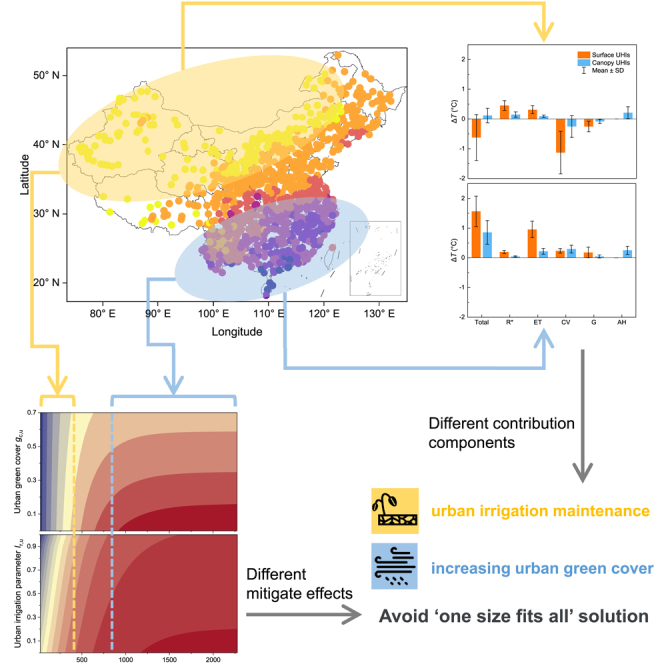

## Introduction

Urban development changes the energy balance in peri-urban areas, which typically have higher air and surface temperatures than their surrounding rural areas.^1^^,^^2^ These well-known urban heat island effects (UHIs) can significantly affect the local climate^1^ and aggravate heat stress,^3^^,^^4^^,^^5^ with negative impacts on energy consumption^6^^,^^7^ and air pollution.^8^^,^^9^^,^^10^ UHIs are also a threat to human health, and a warming environment will increase the risk of morbidity and mortality,^3^^,^^11^ especially when synergistic interactions with heat waves occur.^12^^,^^13^ According to the *World Cities Report 2022: Envisaging the Future of Cities* by UN-Habitat, China’s urban population is expected to exceed 1 billion by 2035, with an estimated increase of 180 million compared with 2020.^14^ China’s rapid urbanization will exacerbate the thermal risks faced by urban populations.^15^^,^^16^ However, one-size-fits-all solutions are frequently employed to mitigate UHIs; another tendency is the conflation between the mitigation strategies of surface UHIs (SUHIs) and canopy UHIs (CUHIs). For instance, shading has a more pronounced cooling effect on surface temperatures than on air temperatures. Therefore, understanding the mechanisms responsible for the UHIs is important for identifying general guidelines to mitigate problems related to heat.

Howard observed higher air temperatures in London compared with surrounding rural areas to first identify the UHIs^17^ and correctly hypothesized most of the causes that are now considered responsible.^1^ However, selecting representative measurement sites for studies of the CUHIs is still a problem that needs to be addressed.^18^^,^^19^ Due to the proliferation of satellite observations in the land surface temperature (LST) field, several studies have attempted to explain the mechanisms responsible for SUHIs.^20^^,^^21^ The same sensor used in a single satellite can provide complete global coverage observation data to obtain large-scale surface temperature observations for applications in comparative studies of cities under different background climatic conditions. However, considering that most vertically oriented building faces are not observed by a nadir-viewing remote imaging radiometer, satellite-based LST data cannot fully represent all of the urban surface temperatures in areas with complex three-dimensional geometry.^22^^,^^23^ In recent studies, the attribution analysis method was applied at city scale to show that the urban–rural contrast in evapotranspiration (*ET*) and/or thermal convection efficiency are the main determinants of summer SUHIs,^24^^,^^25^ and the background climatic conditions affect the relative contributions of these factors.^2^ On a relatively long-period, SUHIs exhibit a tightly connection with CUHIs, with large SUHIs will experience a large CUHIs.^26^ However, observational studies have shown that SUHIs and CUHIs differ in terms of their frequency and characteristics.^27^^,^^28^ Some similar biophysical drivers affect the development of SUHIs and CUHIs, but they may differ in terms of magnitude.^21^ Therefore, the quantitative study on SUHIs in the previous study does not provide a comprehensive understanding of CUHIs; the quantitative attribution of CUHIs remains unclear. Unfortunately, these attribution analysis studies mainly focused on the SUHIs and relatively few have considered the CUHIs.^29^ In particular, compared with the SUHIs, the CUHIs is more relevant to outdoor thermal health^30^ because heat stress is a function of air temperature and not necessarily LST.^31^^,^^32^ Thus, it is important to understand the mechanisms responsible for CUHIs.

In the present study, we focused on urban residential areas where the human activity is more intense. Based on the first-order Taylor series expansion of a linearized energy balance equation, we developed a mechanistic attribution model for linking Δ*T* to net radiation (*R*^∗^), evapotranspiration (*ET*), convection efficiency (*CV*), heat storage (*G*), and anthropogenic heat (*AH*), which used to determine the key factors. This model is based on the fact that during the development of a city, the urban underlying surface changes and coupled with increases in human activity and energy consumption, will lead to change in the original energy balance of this area, and this is considered the main cause of the UHIs. Therefore, background climatic conditions and urban morphology combine to impact the formation of the UHIs. Then, we apply this mechanistic attribution model to discuss the individual and combined effects of background climatic conditions and urban morphology on the formation of SUHIs and CUHIs. The differences in impacts of heat mitigation strategies on SUHIs and CUHIs under different background climatic conditions and their influence by urban morphology were also compared, to provide support for developing general guidelines for urban heat mitigation.

## Results

### Attribution of UHIs

**Figure 1:**
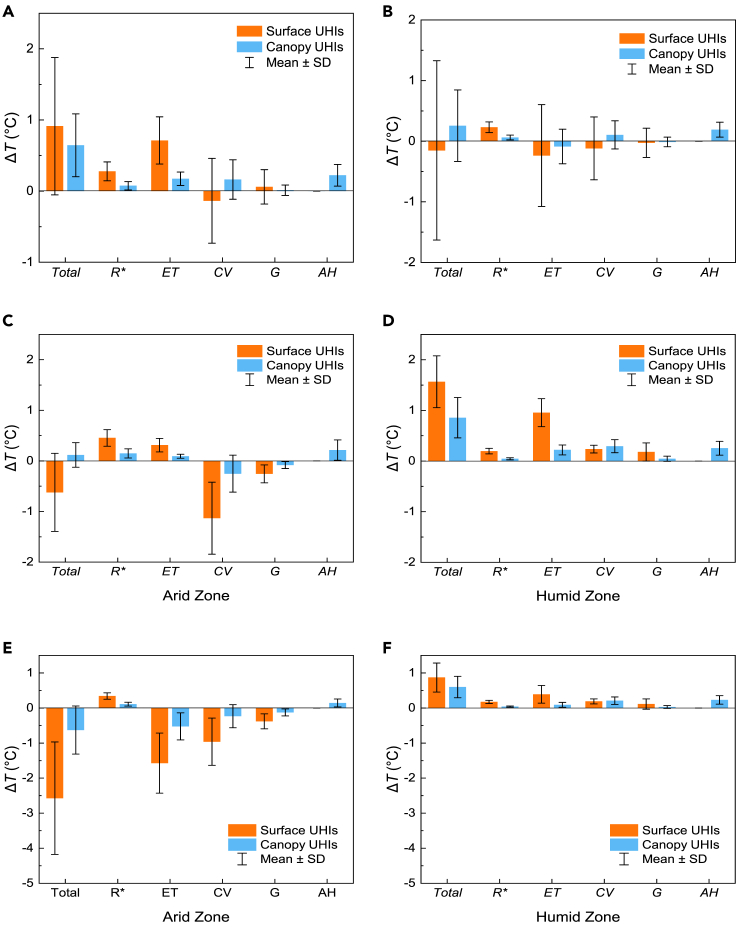
Attributions of summer surface and canopy urban heat islands (UHIs) across China (A and B) Summer UHIs (surface UHIs, orange; canopy UHIs, blue) and components considering urban irrigation indexes of (A) *I*_r,u_ = 0 and (B) *I*_r,u_ = 1. (C and D) Modeling results considering an urban irrigation index of *I*_r,u_ = 0 in arid regions (C) and (D) humid regions. (E and F) Modeling results considering an urban irrigation of index *I*_r,u_ = 1 in (E) arid regions and (F) humid regions. The urban irrigation index is a coefficient describing the level of irrigation, establishing the connection between actual and potential evapotranspiration. For *I*_r,u_ = 0, it describes the actual evapotranspiration under natural background climatic conditions. For *I*_r,u_ = 1, it means there is no water supply limitation and that actual and potential evapotranspiration are equal. Error bars indicate ±1 standard deviation.

Figures 1A and 1B show the attributions to the summer SUHIs and CUHIs intensities for 990 cities across China. Clearly, the main contributors to the summer SUHIs and CUHIs intensities are the urban–rural differences in the evapotranspiration (Δ*ET*) and convection efficiency (Δ*CV*). In particular, when with a low urban irrigation index (*I*_r,u_ = 0), the largest contributor to the SUHI intensities was Δ*ET*, which is consistent with the findings obtained by Li et al.,^24^ and it can be explained by the lower soil moisture and vegetation in urban areas. However, the contribution of Δ*ET* was significantly reduced when the idealized urban irrigation index (*I*_r,u_ = 1) was adopted. Higher *I*_r,u_ will make the evapotranspiration from vegetation in urban areas not be limited by the soil moisture, even with the lower green cover in urban areas, resulting in negative contribution of Δ*ET*, which is more obvious in arid regions. In fact, Δ*ET* also exhibits a large standard deviation (SD), and it is considered that the differences in heat mitigation achieved by urban irrigation are great under different background climatic conditions. Unlike the SUHIs, the difference in the contributions of Δ*ET* and Δ*CV* to the CUHI intensities was not significant. In the present study, the convection efficiency included the convective heat exchange between the canopy air and urban underlying surface, and the convective heat exchange between the canopy air and underlying atmosphere, which is more pronounced for a fluid such as air due to flow. Given the impact of the geometric configuration of an urban canyon on radiation exchanges, the urban canyon receives more net radiation (*R*^∗^) than rural areas,^33^ and the contribution of Δ*R*^∗^ is positive. It is important to note that the difference between urban and rural areas is small, so the contribution of the net radiation is not significant. Meanwhile, the heat stored (*G*) during the daytime was released at nighttime, and the value of *G* was close to 0 when time scales exceed a day, i.e., the contribution of Δ*G* was also small. In addition, the contributions of both Δ*R*^∗^ and Δ*G* to the SUHI intensities were slightly higher than those to the CUHI intensities. It is considered that if the heat stored in air is ignored, *R*^∗^and *G* have direct effects on the urban surfaces and an indirect effect on the urban canopy air, and thus the effect on the CUHI intensities is weaker.

According to the mean annual precipitation (*P*), cities in arid regions (*P* < 400 mm yr^−1^) and humid regions (*P* ≥ 800 mm yr^−1^) were selected to analyze the contributions to the summer SUHI intensities and summer CUHI intensities (see Figures 1C–1F). Δ*T*_s_ ranged from −2.9°C to −0.4°C in arid regions (mean: −0.6°C), and from 0.1°C to 3.2°C in humid regions (mean: 0.9°C). The Δ*T*_s_ values were much smaller for cities in arid regions than humid regions, and the specific contributions to Δ*T*_s_ also differed significantly between arid and humid regions. The average contribution of Δ*R*^∗^ was 0.5°C in arid regions, which was higher than the contribution in humid regions (0.2°C), mainly because the solar radiation is generally higher in arid regions (see Figure S1B). The contributions of Δ*ET* and Δ*CV* were very different in arid and humid regions, and the contribution of Δ*CV* was much higher than that of Δ*ET* in arid regions. Given the short vegetation found in arid regions, cities are generally aerodynamically rougher than the surrounding rural areas and the heat dissipation by convection could be more efficient,^34^ and thus Δ*CV* made a negative contribution to Δ*T*_s_. Urban areas had lower vegetation cover in arid regions, and water limitations reduced the *ET* by vegetation in arid rural areas, thereby limiting the contribution of Δ*ET* to Δ*T*_s_. However, the largest contributor to Δ*T*_s_ was Δ*ET* in urban areas where higher precipitation resulted in a higher soil water content, thereby reducing the restriction on *ET* by vegetation, and the lower vegetation cover in urban areas led to higher Δ*ET* values, and the contribution to Δ*T*_s_ was positive. Cities in humid regions are generally surrounded by tall vegetation, and there was no significant difference in heat dissipation by convection between urban and rural areas, thereby reducing the contribution of Δ*CV* to Δ*T*_s_. The contributions to Δ*T*_c_ were similar to those to Δ*T*_s_, but the differences in the contribution of each factor to Δ*T*_c_ were much smaller compared with Δ*T*_s_. In addition, Δ*CV* provided similar contributions to Δ*T*_s_ and Δ*T*_c_ in humid regions, with values of 0.2°C and 0.3°C under *I*_r,u_ = 0 (0.2°C and 0.2°C under *I*_r,u_ = 1), respectively. Whereas, Δ*CV* provided a significantly greater contribution to Δ*T*_s_ than Δ*T*_c_ in arid regions, with values of −1.1°C and −0.3°C under *I*_r,u_ = 0 and −1.0°C and −0.2°C under *I*_r,u_ = 1, respectively. The contribution of anthropogenic heat (*AH*) was high in both arid and humid regions. Consistent with previous studies, the thermal mitigation effect achieved by increasing the urban irrigation index was influenced by the background climatic conditions.^26^^,^^35^ Increasing the urban irrigation index reduced the contribution of Δ*ET* to Δ*T*_s_ from 0.3°C to −1.6°C in arid regions, but only from 1°C to 0.4°C in humid regions. In addition, the effect of this measure on Δ*T*_c_ was relatively weak, where the contribution of Δ*ET* only decreased from 0.1°C to −0.5°C in arid regions.

### Influence of background climate

**Figure 2:**
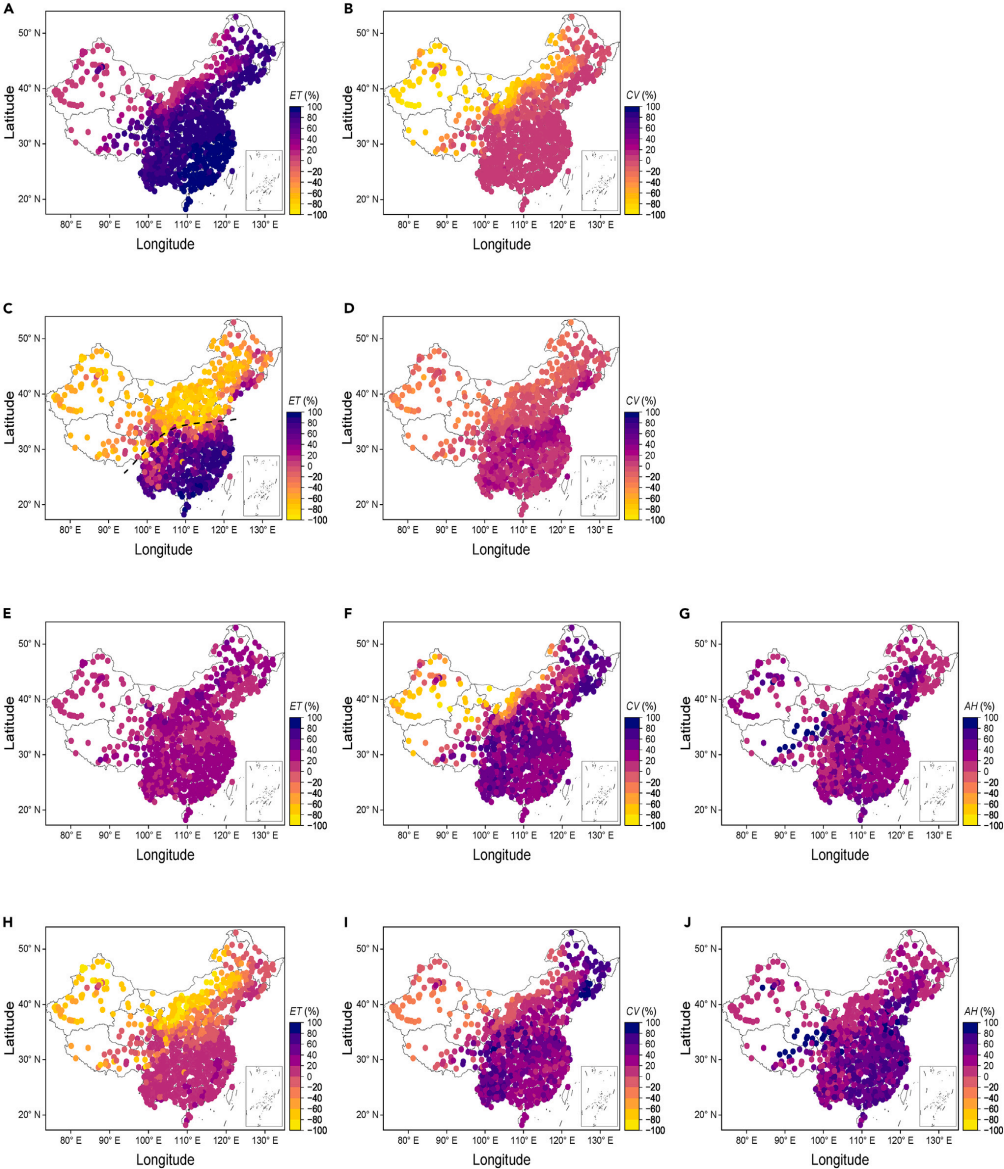
Distribution of the contribution of each component to summer surface and canopy urban heat islands (UHIs) under different urban irrigation indexes (A–D) Surface UHIs and (E–J) canopy UHIs, [(A–B) and (E–G)] under urban irrigation index *I*_r,u_ = 0 and [(C–D) and (H–J)] under urban irrigation index *I*_r,u_ = 1.

We quantified the contributions to Δ*T* from different biophysical factors comprising *R*^∗^, *ET*, *CV*, *G*, and *AH* (see STAR Methods). The distributions of the contributions to Δ*T* in China from different biophysical factors are shown in Figure 2. Without urban irrigation (*I*_r,u_ = 0), the distribution of the contribution from Δ*ET* to Δ*T*_s_ was similar to the distribution for mean annual precipitation in China (see Figure S2). In cities where *P* < 400 mm yr^−1^, the contribution of Δ*ET* was low and generally below 20%, and the contribution to Δ*T*_s_ was dominated by Δ*CV* with greater than −60%, where “–” implies an inverse effect on Δ*T*_s_. Δ*ET* made the dominant contribution to Δ*T*_s_ in humid regions, especially in cities where *P* > 1200 mm yr^−1^, and the contribution of Δ*ET* exceeded 90%. As *I*_ru_ increased, the contribution of *ET* also increased in arid regions, but with the reverse effect on Δ*T*_s_, and this influence gradually expanded from northwest China (arid regions) to southeast China (humid regions). However, in southeast China, *ET* still made a high positive contribution to Δ*T*_s_, especially in areas where *P* > 1600 mm yr^−1^, and Δ*ET* still contributed more than 80%, thereby implying that improving urban irrigation levels alone had a limited impact on mitigating UHIs. It was also found that under idealized urban irrigation (*I*_r,u_ = 1), the contribution of Δ*T*_s_ was more complex in some cities where 800 < *P* < 1200 mm yr^−1^, and both Δ*ET* and Δ*CV* did not make major contributors, and their contributions were similar. As mentioned earlier, the composition of the contributors to Δ*T*_c_ was more complex compared with Δ*T*_s_, and it was difficult to identify the main contributors to Δ*T*_c_, which also suggests that it may be more complex to mitigate Δ*T*_c_ compared with Δ*T*_s_. The dominant contributor was more likely to be identified in arid regions (*P* < 400 mm yr^−1^), where Δ*CV* and Δ*ET* accounted for >60% of the inverse contributions under *I*_r,u_ = 0 and *I*_r,u_ = 1, respectively. Given the influence of latitude, the contribution of Δ*AH* was higher in cities at low latitudes than high latitudes during the summer.

**Figure 3:**
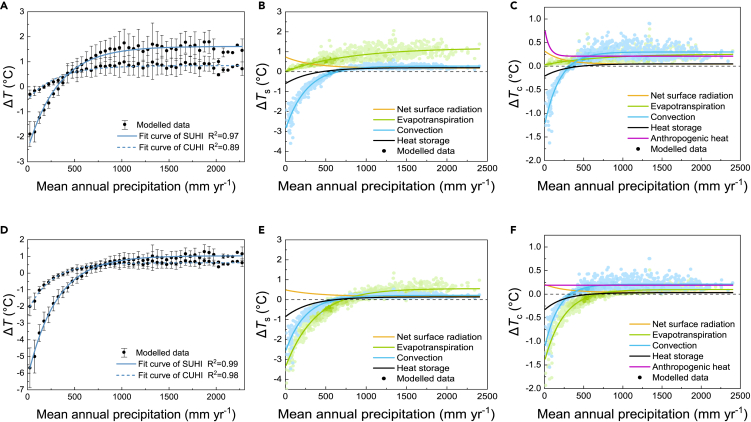
Effects of precipitation on summer surface and canopy urban heat island effects (UHIs) and components (A–C) Urban irrigation index *I*_ru_ = 0. Modeled (markers) and fitted (lines) nonlinear relationships between Δ*T* (Δ*T*_s_ and Δ*T*_c_) and (A) mean annual precipitation (*P*), (B) components of Δ*T*_s_, and (C) components of Δ*T*_c_. (D–F) Urban irrigation index *I*_ru_ = 1. Modeled (markers) and fitted (lines) nonlinear relationships between Δ*T* (Δ*T*_s_ and Δ*T*_c_) and (D) mean annual precipitation, (E) components of Δ*T*_s_, and (F) components of Δ*T*_c_. The line was obtained by fitting the mean values for the modeled data. Error bars indicate ±1 standard deviation.

A nonlinear relationship between Δ*T* (Δ*T*_s_ and Δ*T*_c_) and mean annual precipitation was found (see Figure 3A), which is consistent with the results obtained on a global scale by Manoli et al.^2^ However, compared with their findings, Δ*T*_s_ was slightly lower in the present study because cities in arid regions in China contain high buildings and urban areas have a stronger convective efficiency compared with suburban areas with short vegetation, as shown in Figure 3B. In low precipitation regions, Δ*T*_s_ increased in a linear manner with precipitation, and Δ*T*_s_ saturated at high precipitation values exceeding *P* ≈ 1,000 mm yr^−1^ Δ*T*_c_ shows a similar trend to Δ*T*_s_ but reached saturation at *P* ≈ 500 mm yr^−1^. It was also found that the magnitude of the change in Δ*T*_s_ was much larger than that in Δ*T*_c_, where the latter was influenced less by the background climatic conditions. The *P*–Δ*T* relationship was mainly controlled by Δ*ET* and Δ*CV*, especially Δ*CV*, which had a similar shape to the *P*–Δ*T* relationship. In humid regions with high relative air humidity, the vapor pressure deficit in the air was low, thereby restricting the *ET* by vegetation in rural areas. Thus, an upper bound for Δ*ET* was defined between urban and rural environments. Conversely, water limitations reduced the magnitude of *ET* in rural arid regions, thereby limiting the contribution of Δ*ET* to Δ*T* (see Figures 3B and 3C). Considering the larger “deficit” of water in the air, supplementing the water budget of urban vegetation with irrigation increased the *ET* in urban areas, and it made a negative contribution to Δ*T*, thereby creating an “oasis” effect^36^ (see Figures 3E and 3F). As discussed earlier, the height of natural vegetation increases with precipitation, and thus the heat dissipation efficiency by convection decreased in rural areas as the precipitation increased. Therefore, the urban–rural differences in the convection efficiency also contributed to cooling cities in arid regions.^24^^,^^25^ By contrast, the tall vegetation that surrounded cities in humid regions exchanged heat more efficiently than dense building blocks, and Δ*CV* made a positive contribution to Δ*T*.

The Δ*ET*–Δ*T* and Δ*CV*–Δ*T* relationships had similar shapes for Δ*T*_s_ and Δ*T*_c_. According to Equation 6 and Equation 10, the energy redistribution factors *f*_s_ and *f*_c_ represent the sensitivities of Δ*T*_s_ and Δ*T*_c_ to energy forcing of 1 W m^−2^. As shown in Figure S3, the energy redistribution factor *f*_c_ for Δ*T*_c_ was higher than *f*_s_ for Δ*T*_s_, thereby damping the influence of urban–rural differences in energy forcing on the magnitude of Δ*T*_c_. In addition, to calculate the contribution of Δ*CV* to Δ*T*_c_, the convective heat exchange between the urban surface and canopy air was also considered, which generally had a positive influence. Therefore, this reduced the negative influence of Δ*CV* on Δ*T*_c_ in arid regions, whereas Δ*CV* had a greater positive influence on Δ*T*_c_ than Δ*T*_s_ in humid regions. It was also found that *f*_s_ and *f*_c_ varied with *P*, which tended to be higher in humid regions (see Figure S3). Thus, temperatures were more stable in humid areas and less susceptible to energy forcing, which may also explain the difficulty mitigating UHIs in these cities because it is necessary to eliminate more heat fluxes.

### Influence of urban morphology

**Figure 4:**
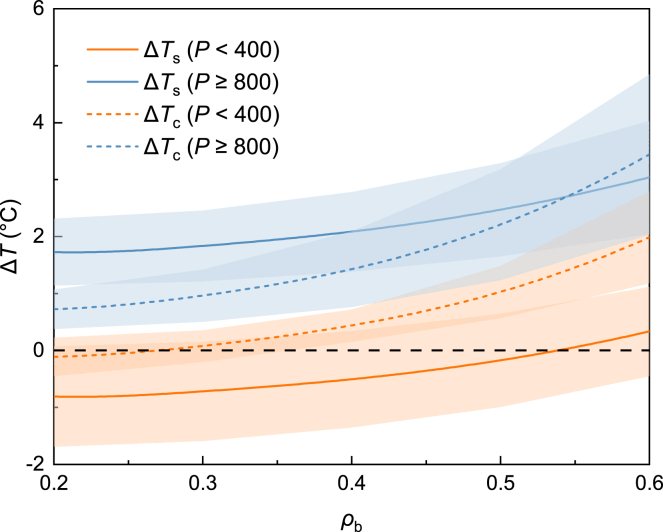
Effects of different building densities on the urban heat island effect in arid (orange) and humid regions (blue) Solid lines (Δ*T*_s_) and dashed lines (Δ*T*_c_) indicate the ensemble means, and shaded areas represent the ensemble mean ±1 standard deviation.

**Figure 5:**
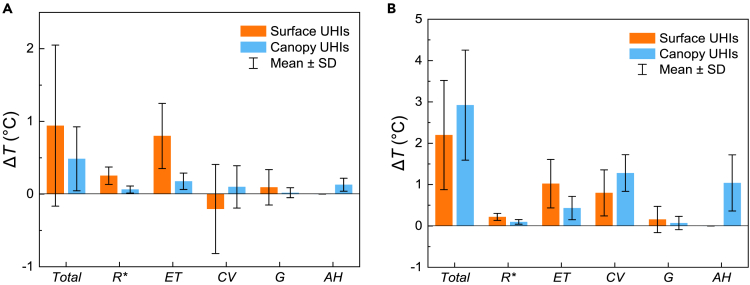
Attribution of summer surface and canopy urban heat island effects (UHIs) under different building densities Summer UHIs (surface UHIs, orange; canopy UHIs, blue) and their components at: (A) building density *ρ*_b_ = 0.2 and (B) building density *ρ*_b_ = 0.6. Error bars indicate ±1 standard deviation.

We selected a building density (*ρ*_b_) range of 0.2–0.6 to analyze the influence of differences in urban morphology on the UHIs in arid and humid regions. In contrast to urban green cover, the influence of urban morphology on the UHIs was affected less by the background climatic conditions. In arid and humid regions, the Δ*T*–*ρ*_b_ relationship had a similar nonlinear shape, and Δ*T*_c_ was influenced more significantly by the building density compared with Δ*T*_s_ (see Figure 4). The attribution of UHI intensities for building densities of 0.2 and 0.6 were analyzed (see Figure 5). Under a building density of 0.2, Δ*T*_s_ was slightly higher than Δ*T*_c_, whereas the opposite was found with the building density of 0.6. The dominant contributor to Δ*T*_s_ was Δ*ET* in both the cases, and the different contribution of Δ*ET* to Δ*T*_s_ was also influenced by background climatic conditions, which shows more significance in high precipitation regions (see Figure S7A). Δ*ET* also shows a similar contribution trend to Δ*T*_c_, but the contributions of Δ*ET*, Δ*CV*, and Δ*AH* to Δ*T*_c_ were all around 0.15°C, with no significant dominant contributor (*ρ*_b_ = 0.2, see Figure 5A). The contribution of Δ*CV* to Δ*T*_s_ and Δ*T*_c_ still cannot be ignored and increases significantly with increasing building density, especially for Δ*T*_c_. In contrast to Δ*ET*, the different contribution of Δ*CV* to Δ*T*_s_ or Δ*T*_c_ is more significant in arid regions, whereas the contribution to Δ*T*_c_ is more susceptible to the influence of building density compared with Δ*T*_s_ (see Figure S7). It is suggested that as the building density increased, higher building densities restricted the efficiency of convective heat transfer from the urban surface and urban canopy to the atmosphere, thereby resulting in poorer heat dissipation with a negative impact on the UHIs. The higher building density also led to more anthropogenic heat^37^^,^^38^ and when coupled with the poor heat dissipation, the contribution of Δ*AH* to Δ*T*_c_ was significant at a building density of 0.6 (see Figure 5B). Therefore, maintaining lower building densities is beneficial for urban heat mitigation, which provides greater heat dissipation and space for urban greening. It needs to be clarified that as the urban population is growing, choosing higher buildings or building densities to accommodate urban residents still requires further research, because the exact relation between aerodynamic resistance and urban morphology is complicated.^24^ The urban morphology should be able to enhance heat dissipation performance.

### Heat mitigation strategies

**Figure 6:**
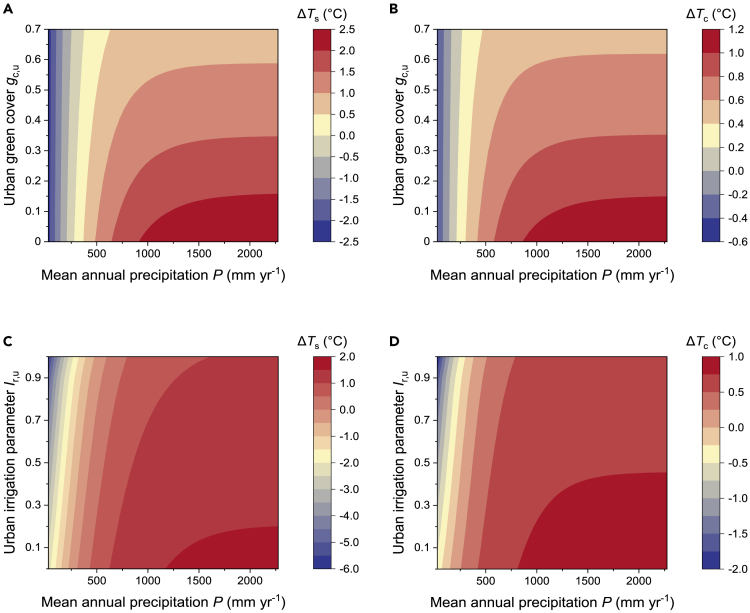
Impact of urban background climate on the efficiency of heat mitigation by urban evapotranspiration (A and B) Mitigation effect of urban green cover change on (A) Δ*T*_s_ and (B) Δ*T*_c_. (C and D) Mitigation effect of urban irrigation changes on (C) Δ*T*_s_ and (D) Δ*T*_c_. Results are shown for *I*_r,u_ = 0.2 in (A–B) and for constant green cover *g*_c,u_ = 0.3 in (C–D).

Urban greenery is used as a strategy for mitigating the UHIs, and it plays an important role in promoting the urban outdoor thermal environment as well as enhancing the thermal comfort of residents.^39^^,^^40^ It shows that the *g*_c,u_–Δ*T* relationship was strongly influenced by the background climatic conditions in urban areas (see Figures 6A and 6B). In humid regions, the *ET* by vegetation is less water limited, and it is a dominant component of the surface energy balance in rural areas.^41^ The urban–rural *ET* difference was significant due to the lack of vegetation in cities, and thus increased vegetation cover is needed to reduce Δ*T*_s_ and Δ*T*_c_. This means that it is difficult to reduce Δ*T*_s_ and Δ*T*_c_ by only focusing on vegetation strategies in higher precipitation regions (*P* > 1000 mm yr^−1^), because the growth of vegetation is strongly dependent on the precipitation and humidity.^42^ Indeed, to achieve Δ*T*_s_ < 1°C, almost the entire city area would need to be replaced with vegetation, and it is almost impossible to achieve Δ*T*_s_ < 0.5°C without urban irrigation management (*I*_r,u_ = 0). The *g*_c,u_–Δ*T* relationship had a similar shape for Δ*T*_s_ and Δ*T*_c_, but with a lower value of Δ*T*_c_, it is more feasible to achieve a weak Δ*T*_c_ by applying urban greenery strategies. In addition to increasing urban green cover, improving urban irrigation is an effective method for achieving urban cooling (see Figures 6C and 6D). In particular, the low soil moisture content in arid regions will limit the evapotranspiration by rural vegetation to a certain extent.^2^ Therefore, increasing urban irrigation can effectively increase the soil moisture content and reduce the limitation on vegetation evapotranspiration, achieving high heat mitigation even with low urban green cover (*g*_c,u_ = 0.3). A recent study has shown that urban irrigation in more arid areas can improve the potential cooling performance.^43^ By contrast, in humid regions with high soil moisture contents, the evapotranspiration by vegetation is less restricted by water, and thus the heat mitigation effect achieved by increasing urban irrigation is weaker (see Figure S10).

Clearly, increasing urban green cover and urban irrigation are both feasible strategies for mitigating the heat island effect.^44^^,^^45^ In addition, according to further analysis, the effects achieved by these two measures also differed among different background climatic conditions. Urban green cover of 0 was set as a base case, and it was considered the UHI mitigation effect is achieved by increasing urban green cover under different urban irrigation indexes (see Figure S8). Under a low urban irrigation index (*I*_r,u_ = 0.3), the heat island mitigation effect achieved by increasing urban green cover was more significant in humid regions, and this effect saturated at high precipitation values exceeding *P* ≈ 1000 mm yr^−1^ (see Figure S8B). Interestingly, the UHI mitigation effect decreased with increasing precipitation in arid regions (*P* < 400 mm yr^−1^). It is suggested that the difference between the potential evapotranspiration (*PET*) and mean annual precipitation decreased rapidly in this range (*P* < 400 mm yr^−1^) (see Figure S9), and this difference indicates a contradiction between the positive effect of ambient air moisture deficit on vegetation evapotranspiration and the limitation imposed on vegetation evapotranspiration by the lack of available water. Therefore, the results suggested that urban irrigation management was more important in arid regions, even with a low urban irrigation index (*I*_r,u_ = 0.3), where the water provided by irrigation could promote the evapotranspiration by vegetation, and the UHI mitigation effect decreased as the precipitation increased. By contrast, with no urban irrigation management (*I*_r,u_ = 0), the evapotranspiration by vegetation was affected by the availability of water, and the situation mentioned earlier did not occur, and the UHI mitigation effect increased with the precipitation. In addition, significant differences in the heat island mitigation effects were found as the urban green cover increased in humid regions (see Figure S8A). The opposite situation occurred under a higher urban irrigation index (*I*_r,u_ = 1), where the heat island mitigation effect of greater urban green cover increased in a nonlinear manner as the precipitation increased, before saturating at precipitation of *P* ≈ 500 mm yr^−1^, which is consistent with the results shown in Figure S9 to further illustrate the importance of urban irrigation management in arid regions (see Figure S10). The mitigation effect was much lower for Δ*T*_c_ than Δ*T*_s_, i.e., about half of that for Δ*T*_s_, and both exhibited similar trends. This can be explained by the higher energy redistribution factor of Δ*T*_c_ than Δ*T*_s_.

The effectiveness of heat mitigation strategies is influenced not only by background climatic conditions but also by urban morphologies. The Mann-Whitney U test was used to analyze whether the heat mitigation with different building density had a significant difference, as shown in Figures S11A and S11B. The heat mitigation strategy obtains more significant effects at the higher building density (p < 0.01) and shows more difference in reducing Δ*T*_c_ than Δ*T*_s_. At *I*_r,u_ = 0.2, the reduction of Δ*T*_s_ and Δ*T*_c_ by increasing urban green cover from 0 to 0.4 was more significant in humid regions, even at a building density of 0.6 (see Figure S11C). It needs to be clarified that although the heat mitigation effect of increasing urban green cover is weaker at *ρ*_b_ = 0.2 than at *ρ*_b_ = 0.6, more space can be provided for urban greening at *ρ*_b_ = 0.2, which can further reduce Δ*T*_s_ and Δ*T*_c_. In arid regions, increasing the urban irrigation index to reduce Δ*T*_s_ and Δ*T*_c_ was more favorable, and the urban morphology still shows a significant effect on reducing Δ*T*_c_ compared with Δ*T*_s_ (see Figure S11D). Fortunately, heat mitigation strategies can still have significant effects at higher building densities under different background climatic conditions, which of course requires a combination of urban greening and management of urban irrigation. It needs to be clarified that heat mitigation strategies will increase air humidity, especially in humid regions, which may further increase heat risk.^46^

## Discussion

In the UHI biophysical mechanism model, the difference between the urban and rural surface temperature (Δ*T*_s_) and air temperature (Δ*T*_c_) is attributed to different biophysical factors, thereby improving the understanding of the impacts of energy balance changes caused by urbanization on the LST and air temperature. In the present study, it is demonstrated that the urban–rural differences in evapotranspiration (Δ*ET*) and the convection efficiency (Δ*CV*) made the two main contributions to Δ*T*_s_. Considering that anthropogenic heat release will directly cause air temperature increases, anthropogenic heat flux also had an important effect on Δ*T*_c_, thereby making the mitigation of Δ*T*_c_ more complicated. It needs to be clarified that the air temperature rise due to anthropogenic heat release will also play an indirect effect on the surface temperature by limiting the release of heat from the urban surface. However, it has been shown to play a negligibly small role in contributing to Δ*T*_s_^2^, so we do not consider it as a factor affecting Δ*T*_s_. Urban background climatic conditions significantly influenced the components that contributed to Δ*T*_s_ and Δ*T*_c_, and the relationship between Δ*T* and mean annual precipitation was nonlinear.

Anthropogenic heat release will directly cause air temperature increases, which also had an important effect on Δ*T*_c_, especially at the higher building density. Furthermore, the energy redistribution factor of Δ*T*_c_ (*f*_c_) is generally higher than that of Δ*T*_s_ (*f*_s_), which means it is necessary to eliminate more heat fluxes to reducing Δ*T*_c_. Therefore, the factors that contribute to Δ*T*_c_ are more complex than Δ*T*_s_, and it may be more challenging to mitigate Δ*T*_c_. Maintaining a low building density should be prioritized, especially when mitigating Δ*T*_c_, which can provide more space for urban greenery and further reduce anthropogenic heat emissions.

Increasing urban green cover is an important strategy for mitigating the UHIs,^44^^,^^47^ and the effect of this strategy was influenced by the background climatic conditions.^35^^,^^47^ Without urban irrigation, the effect of increasing urban green cover on mitigating UHIs was more pronounced in humid regions, but mitigation was difficult to achieve with a lower Δ*T*_s_ and Δ*T*_c_. In arid regions, it is particularly important to improve urban irrigation considering the limitations imposed by soil water deficit on evapotranspiration. It is important for mitigating the UHIs, and a highly significant mitigation effect can be obtained by increasing urban irrigation even with low urban green cover. However, the irrigation strategy might jeopardize scarce water resources when considering the urban heat mitigation.^48^ Meanwhile, urban greening and urban irrigation may lead to elevated humidity levels, which is also an important contributor to human heat stress.^46^ Although urban greenery can effectively reduce Δ*T*_c_, its mitigation effect was much lower than Δ*T*_s_, about half of that for Δ*T*_s_.

The contributions of urban–rural heat differences in the convection efficiency to Δ*T*_s_ and Δ*T*_c_ are more complicated to assess. Because the exact relation between aerodynamic resistance and urban morphology is complicated, it is difficult to artificially change the efficiency of urban convection. Generally, the positive impact of Δ*CV* on Δ*T*_c_ was higher than that on Δ*T*_s_, at different building densities. Thus, improving urban convective heat exchange efficiency plays more significant role in mitigating Δ*T*_c_ than mitigating Δ*T*_s_, which means maintaining a low building density is most important for mitigating Δ*T*_c_. Improving urban convective heat exchange efficiency may be more important in those cities with water limitations by changing the urban morphology.^21^ Unfortunately, this heat mitigation strategy will face more challenges, as their exact relations are complicated.^24^ This heat mitigation strategy was difficult to use in built-up neighborhoods where buildings already exist. For new neighborhoods, rational urban morphology design can be considered to improve urban convective heat exchange efficiency.

### Limitations of the study

Generally, improving the urban evapotranspiration capacity and urban convective heat exchange efficiency plays an important role in mitigating Δ*T*_s_ and Δ*T*_c_.^2^^,^^24^ In arid regions, it is more important to improve urban irrigation than increasing the urban green cover. By contrast, in humid areas, it is quite important to maintain high urban green cover in urban areas to reduce the negative impacts of urban–rural differences in evapotranspiration on Δ*T*_s_ and Δ*T*_c_ considering the strong evapotranspiration capacity in rural areas.^2^ However, the present model does not consider the effects of impervious surfaces on the urban water balance (the soil moisture may be lower in urban areas than suburban areas) but ensuring adequate urban irrigation is important. Urban morphology also affects the effectiveness of heat mitigation strategies. Targeted solutions should be proposed for different neighborhoods of the same city, especially in humid regions, where the impact of differences in urban morphology was more obvious. Furthermore, our discussion is limited to the summer-averaged SUHIs and CUHIs; some specific climatic conditions such as large-scale and local circulation, especially for the synergistic effect with heat waves in summer, are not considered. These factors can influence physical mechanisms that drive the SUHIs and CUHIs, as well as the efficacy of heat mitigation strategies.^49^^,^^50^ In-depth and extensive studies are required to elucidate the general principles.

## STAR★Methods

### Key resources table

**Table:** 

REAGENT or RESOURCE	SOURCE	IDENTIFIER
**Background meteorological data**		

Typical meteorological year data	China building energy efficiency design basic data platform	https://buildingdata.xauat.edu.cn/
	EnergyPlus	https://energyplus.net/weather
Mean annual precipitation data	China Meteorological Data Network	http://data.cma.cn/

### Resource availability

#### Lead contact

Requests for further information and resources should be directed to the lead contact, Liu Yan (liuyan@xauat.edu.cn).

#### Materials availability

This study did not generate new materials.

#### Data and code availability

All data can be obtained from the lead contact, provided the request is reasonable.

The code related to the attribution model can be accessed by reaching out to the lead contact.

### Methods details

#### Urban morphology

The present study focused on the thermal environment in residential areas at the neighborhood scale (≤ 1 km). It is generally assumed that megacities contain more tall buildings but according to a recent study of 36 major cities in China, the mean building height at the city level is not related to the city size, and the urban morphology parameters (building height, building width, and street width) are similar in residential areas.^51^ At the neighborhood scale, various urban morphologies are present in Chinese cities, and urban morphology also has a significant impact on the urban thermal environment.^52^ Therefore, when analyzing the impact of background climatic conditions on UHIs, the influence generated by changes in urban morphology was eliminated by selecting the common urban morphology as the base case for the model. Additionally, the impact of urban morphology on UHIs has also been studied, we selected combinations of different urban morphology parameters. According to the building height data set for China, the average building height is generally below 20 m.^51^^,^^53^ Beijing City Lab (https://www.beijingcitylab.com/) provides data for 141375 street blocks in 63 Chinese cities during 2017, with three-dimensional parameters at the street block scale for these Chinese cities. According to this data set, the average number of building stories is predominantly <7 in China, which is also consistent with the results obtained in a previous study,^51^^,^^53^ and the building density is mainly medium and high density (*ρ*_b_ > 0.25) in these street blocks. Therefore, the urban morphology parameters established for the base case in the study comprised: building height *h*_b_ = 18 m (about 6 stories), building density *ρ*_b_ = 0.3, building width *w*_b_ = 20 m,^54^ and *g*_c,u_ = 0.3. We also considered the effects of differences in urban morphology by changing the building density, in the range of 0.2–0.6, based on the street block data from 63 Chinese cities provided by the Beijing City Lab, which shows most building densities in China ranged from 0.22 to 0.52.

#### Background climate

The climate conditions vary significantly among years and the background climate data for a given year do not reflect the general UHIs in a city. Thus, it is required a customized background climate data set that adequately reflected the typical background climatic conditions in cities, so we obtained typical meteorological year data from the China building energy efficiency design basic data platform (https://buildingdata.xauat.edu.cn/) as background meteorological data. Typical meteorological year data for Macau and Taipei were obtained from EnergyPlus (https://energyplus.net/weather). Mean annual precipitation data were retrieved from the 1970–2017 monthly precipitation data set of the China Meteorological Data Network (http://data.cma.cn/).

##### Mathematical model

Multivariable functions of *T*_s_ (surface temperature) and *T*_c_ (air temperature) were derived from the energy balance in rural or urban areas. Next, based on the first-order Taylor series expansion, the mechanistic attribution model for attributing the UHI intensity to contributions from different factors was developed (radiation, evapotranspiration, convection efficiency, heat storage, and anthropogenic heat).^2^^,^^24^^,^^25^ The surface energy balance model validation was performed with the 2013 Global Urban Heat Island Dataset, which was provided by Manoli et al.^2^ We selected cities with populations over 10^5^ for model validation. The observed urban surface temperature was obtained from the observed rural surface temperature + urban heat island intensity (i.e. Ts_summer_2013 + dTs_summer_2013). Comparison of the surface temperature observed and simulated in rural and urban areas were shown in Figures S12A and S12B, respectively. The surface energy balance model shows higher accuracy in rural areas (rural: RMSE = 1.0°C, urban: RMSE = 4.3°C), due to the more complex urban underlying surface in urban areas. The canopy energy balance model validation was performed with the observed data in Xi’an, China, in 2016, which available from China building energy efficiency design basic data platform (https://buildingdata.xauat.edu.cn/). The hourly meteorological data are averaged to obtain daily data for the model inputs. Comparison of the canopy air temperature observed and simulated in rural and urban areas were shown in Figures S13A and S13B, respectively. Similar to the surface energy balance model, the canopy energy balance model shows higher accuracy in rural areas (rural: RMSE = 0.1°C, urban: RMSE = 1.0°C). Considering the error caused by the first-order Taylor series expansion of the mechanistic attribution model, the mean values of the rural and urban variables were used as parameters in this model, the mechanistic attribution model validation as shown in Figures S12C and S13C.

#### Mechanistic attribution model

Based on the first-order Taylor series expansion, we attributed the UHI intensity (including SUHIs and CUHIs) to contributions from different biophysical factors. For SUHIs, based on the surface energy balance equation:

(Equation 1) $$Q^{*}=S^{*}+L^{*}=Q_{H}+Q_{E}+Q_{G}$$

where *Q*^∗^ is the net surface radiation [W·m^–2^], *S*^∗^ is the net short-wave radiation [W·m^–2^], i.e., $S^{*}=S_{\text{in}}\left(1-\alpha \right)$, *S*_in_ is the incoming short-wave radiation [W·m^–2^], *α* is the surface albedo, and *L*^∗^ is the net long-wave radiation [W·m^–2^], i.e., $L^{*}=L_{\text{in}}-L_{\text{out}}$, where *L*_in_ is the incoming long-wave radiation [W·m^–2^]. Considering that these data were missing for many areas, we modeled $L_{\text{in}}=\varepsilon_{a}\sigma {T_{a}}^{4}$^55^, where *ε*_a_ is the atmospheric emissivity, i.e., $\varepsilon_{a}=1.723{\left(\frac{e_{\text{sat}}\left(T_{a}\right)\frac{RH}{100}}{T_{a}}\right)}^{\frac{1}{7}}$, *e*_sat_(*T*_a_) is the saturation vapor pressure at temperature *T*_a_ [kPa], i.e., $e_{\text{sat}}\left(T_{a}\right)=0.611\;\exp\left(\frac{17.27\left(T_{a}-273.15\right)}{T_{a}-35.85}\right)$^56^, *σ* is the Stefan–Boltzmann constant (*σ* = 5.67 × 10^–8^ W·m^–2^·K^–4^), and *T*_a_ is the air temperature which measurement at the rural weather station [K]. In addition, *L*_out_ is the upward long-wave radiation [W·m^–2^], i.e., $L_{\text{out}}=\varepsilon_{s}\sigma {T_{s}}^{4}+\left(1-\varepsilon_{s}\right)L_{\text{in}}$, where *ε*_s_ is the surface emissivity and *T*_s_ is the LST [K]. *Q*_H_ is the sensible heat flux [W·m^–2^], i.e., $Q_{H}=\frac{\rho c_{p}\left(T_{s}-T_{a}\right)}{r_{a}}$, where *ρ* is the air density [kg·m^–3^], *c*_p_ is the specific heat of air at constant pressure [J·kg^–1^·K^–1^], and *r*_a_ is the aerodynamic resistance [s·m^–1^]. *Q*_E_ is the latent heat flux [W·m^–2^], i.e., $Q_{E}=g_{c}\beta \frac{\rho l_{v}\left[q_{\text{sat}}\left(T_{s}\right)-q_{a}\right]}{r_{a}+r_{s}}$^2^, where *g*_c_ is the green cover, *β* is the water stress factor, *l*_v_ is the latent heat of vaporization [J·kg^–1^], and *q*_sat_(*T*_s_) is the saturated specific humidity at temperature *T*_s_ [kg·kg^–1^], i.e., $q_{\text{sat}}\left(T_{s}\right)=0.622\frac{e\text{sat}\left(Ts\right)}{p\text{atm}-0.378e\text{sat}\left(Ts\right)}$, where *p*_atm_ is the atmospheric pressure [kPa], *q*_a_ is the specific humidity of air [kg·kg^–1^], and *r*_s_ is the surface resistance [s·m^–1^]. *Q*_G_ is the heat storage [W·m^–2^], which can be modeled by the objective hysteresis model (OHM). In this surface energy balance equation, we considered the heat exchange between the urban surface and the atmosphere (*Q*_H_), while excluding the impact of urban canopy air temperature. Subsequently, we obtain the surface temperature as a boundary condition for the canopy energy balance equation (*Q*_s_). Meanwhile, the anthropogenic heat flux directly affects air in the urban canopy to increase the air temperature, which then indirectly affects the heat dissipation from the urban surface, so we considered the effect of anthropogenic heat flux in the canopy energy balance equation (see the next section).

To obtain an analytical form of *T*_s_, the outgoing long-wave radiation term and the saturated specific humidity term were linearized at the point *T*_a_:

(Equation 2) $$\varepsilon_{s}\sigma {T_{s}}^{4}\approx \varepsilon_{s}\sigma {T_{a}}^{4}+4\varepsilon_{s}\sigma {T_{a}}^{3}\left(T_{s}-T_{a}\right)$$

(Equation 3) $$q_{\text{sat}}\left(T_{s}\right)\approx q_{\text{sat}}\left(T_{a}\right)+q{{\prime_{\text{sat}}}_{,}}_{s}\left(T_{a}\right)\left(T_{s}-T_{a}\right)$$

where *q'*_sat,s_(*T*_a_) is the derivative of *q*_sat_(*T*_a_) relative to *T*_a_.

By substituting Equations 2 and 3 into Equation 1 and rearranging, built the following analytical solution for *T*_s_.

(Equation 4) $$T_{s}=\frac{S_{\text{in}}\left(1-\alpha \right)+\varepsilon_{s}\sigma \left(\varepsilon_{a}-1\right){T_{a}}^{4}-g_{c}\beta \frac{\rho l_{v}\left[q_{\text{sat}}\left(T_{a}\right)-q_{a}\right]}{r_{a}+r_{s}}-Q_{G}}{4\varepsilon_{s}\sigma {T_{a}}^{3}+\frac{\rho c_{p}}{r_{a}}+g_{c}\beta \frac{\rho l_{v}}{r_{a}+r_{s}}q_{\text{sat}}^{\prime }\left(T_{a}\right)}+T_{a}$$

In this study, urbanization was considered as a perturbation of the rural base state and only consider changes in LST due to changes in five biophysical factors (i.e., Δ*α*, Δ*ε*_s_, Δ*r*_a_, Δ*r*_s_, and Δ*Q*_G_). According to Taylor’s theorem, the urban–rural LST difference was expressed as:

(Equation 5) $$\Delta T_{s}=\frac{1}{f_{s}}\left\{\begin{array}{l} -S_{\text{in}}\Delta \alpha +\sigma \left(\varepsilon_{a}{T_{a}}^{4}-{T_{s}}^{4}\right)\Delta \varepsilon_{s}+\left[\frac{\rho c_{p}}{{r_{a}}^{2}}\left(T_{s}-T_{a}\right)+g_{c}\beta \frac{\rho lv\left[q_{\text{sat}}\left(T_{s}\right)-q_{a}\right]}{{\left(r_{a}+r_{s}\right)}^{2}}\right]\Delta r_{a}\\ +\beta \frac{\rho lv\left[q_{\text{sat}}\left(T_{s}\right)-q_{a}\right]}{r_{a}+r_{s}}\Delta g_{c}-\Delta Q_{G} \end{array}\right\}$$

(Equation 6) $$f_{s}=4\varepsilon_{s}\sigma {T_{s}}^{3}+\frac{\rho c_{p}}{r_{a}}+g_{c}\beta \frac{\rho l_{v}}{r_{a}+r_{s}}q_{\text{sat}}^{\prime }\left(T_{s}\right)$$

where *f*_s_^–1^ represents the sensitivity of LST to changes of 1 W·m^–2^ in energy forcing at the land surface.^2^^,^^57^

Similar to SUHI, CUHI is based on the canopy energy balance equation:

(Equation 7) $$Q_{c}=Q_{s}+Q_{\text{ah}}$$

where *Q*_c_ denotes turbulent exchanges between the air temperature in the urban canyon and atmosphere [W·m^–2^], i.e., $Q_{c}=\frac{\rho c_{p}\left(T_{c}-T_{\text{atm}}\right)}{r_{a}}$^58^; *T*_atm_ is the temperature of the atmosphere above the urban canopy [K], and considering that these data were missing for many cities, which was assumed that it was equal to the air temperature measured at the rural weather station, i.e., *T*_atm_ = *T*_a_; *T*_c_ is the canopy air temperature [K], *Q*_s_ denotes turbulent exchanges between the air temperature in the urban canyon and urban surface [W·m^–2^], i.e., $Q_{s}=\rho c_{p}C_{h}\left(T_{s}-T_{c}\right)$, *C*_h_ denotes the turbulent transfer coefficient for sensible heat [s·m^–1^], and *Q*_ah_ is the anthropogenic heat flux [W·m^–2^] calculated based on the building energy model framework.^59^ In residential buildings, the heat load generated by indoor human metabolism and heat dissipation from equipment (electric lights, etc.) is relatively small relative to the overall anthropogenic heat, so only considered the heat load generated by heat transfer through the building structure and ventilation into the room^60^: $Q_{\text{ah}}=\left(H_{\text{out}}+E_{\text{out}}\right)\frac{1+COP}{COP}$, where *H*_out_ and *E*_out_ are the sensible and latent heat pumped out from the building, respectively, and *COP* represents the energy efficiency of heat source equipment. $H_{\text{out}}=\left[h\left(T_{s}-T_{\text{in}}\right)+\left(1-\beta_{\text{in}}\right)\rho c_{p}v_{a}\left(T_{c}-T_{\text{in}}\right)\right]\varphi_{p}$, where *h* is the convective heat transfer coefficient [W·m^–2^·K^–1^], *T*_in_ is the indoor air temperature [K], which was selected according to the local air temperature, *β*_in_ is the thermal efficiency of the total heat exchanger, *v*_a_ is the total ventilation rate in the building [m^3^·h^–1^], and *φ*_p_ is the ratio of hourly occupants relative to the peak number of occupants per floor area (0 ≤ *φ*_p_ ≤ 1). $E_{\text{out}}=\left(1-\beta_{\text{in}}\right)l_{v}\rho v_{a}\left(q_{a}-q_{\text{in}}\right)\varphi_{p}$, where *q*_in_ is the specific humidity of the indoor air [kg·kg^–1^].

By rearranging the canopy energy balance equation, built the following analytical solution for *T*_c_.

(Equation 8) $$T_{c}=\frac{\frac{\rho c_{p}T_{\text{atm}}}{r_{a}}+\rho c_{p}C_{h}T_{s}+Q_{\text{ah}}}{\frac{\rho c_{p}}{r_{a}}+\rho c_{p}C_{h}}$$

Similar to SUHI, according to Taylor’s theorem, the urban–rural air temperature difference was expressed as:

(Equation 9) $$\Delta T_{c}=\frac{1}{f_{c}}\left[\rho c_{p}C_{h}\Delta T_{s}+\frac{\rho c_{p}}{r_{a}^{2}}\left(T_{c}-T_{\text{atm}}\right)\Delta r_{a}+\rho c_{p}\left(T_{s}-T_{c}\right)\Delta C_{h}+\Delta Q_{\text{ah}}\right]$$

(Equation 10) $$f_{c}=\frac{\rho c_{p}}{r_{a}}+\rho c_{p}C_{h}$$

where *f*_c_^–1^ represents the sensitivity of the air temperature to changes of 1 W·m^–2^ in energy forcing in the urban canyon.

The contributions to Δ*T* from different biophysical factors comprising *R*^∗^, *ET*, *CV*, *G*, and *AH* were expressed as:

(Equation 11) $$Contribution_{i}=\frac{\Delta i\left|\Delta i\right|}{{\left(\Delta R^{*}\right)}^{2}+{\left(\Delta ET\right)}^{2}+{\left(\Delta CV\right)}^{2}+{\left(\Delta G\right)}^{2}+{\left(\Delta AH\right)}^{2}}\times 100\%$$

where *Contribution*_*i*_ denotes the contribution of a particular biophysical factor, *i* = {*R*^∗^, *ET*, *CV*, *G*, *AH*}.

(Equation 12) $$\Delta R^{*}=\left\{ \begin{array}{cc} \frac{1}{f_{s}}\left(-S_{\text{in}}\Delta \alpha +\sigma \left(\varepsilon_{a}{T_{a}}^{4}-{T_{s}}^{4}\right)\Delta \varepsilon_{s}\right) & \text{in}\;\Delta T_{s}\\ \frac{1}{f_{s}}\frac{1}{f_{c}}\rho c_{p}C_{h}\left(-S_{\text{in}}\Delta \alpha +\sigma \left(\varepsilon_{a}{T_{a}}^{4}-{T_{s}}^{4}\right)\Delta \varepsilon_{s}\right) & \text{in}\;\Delta T_{c} \end{array}\right.$$

(Equation 13) $$\Delta ET=\left\{ \begin{array}{cc} \frac{1}{f_{s}}\left(\beta \frac{\rho l_{v}\left[q_{\text{sat}}\left(T_{s}\right)-q_{a}\right]}{r_{a}+r_{s}}\Delta g_{c}\right) & \text{in}\;\Delta T_{s}\\ \frac{1}{f_{s}}\frac{1}{f_{c}}\rho c_{p}C_{h}\left(\beta \frac{\rho l_{v}\left[q_{\text{sat}}\left(T_{s}\right)-q_{a}\right]}{r_{a}+r_{s}}\Delta g_{c}\right) & \text{in}\;\Delta T_{c} \end{array}\right.$$

(Equation 14) $$\Delta CV=\left\{ \begin{array}{cc} \frac{1}{f_{s}}\left(\left[\frac{\rho c_{p}}{{r_{a}}^{2}}\left(T_{s}-T_{a}\right)+g_{c}\beta \frac{\rho lv\left[q_{\text{sat}}\left(T_{s}\right)-q_{a}\right]}{{\left(r_{a}+r_{s}\right)}^{2}}\right]\Delta r_{a}\right)\; & \text{in}\;\Delta T_{s}\\ \frac{1}{f_{s}}\frac{1}{f_{c}}\rho c_{p}C_{h}\left(\beta \frac{\rho l_{v}\left[q_{\text{sat}}\left(T_{s}\right)-q_{a}\right]}{r_{a}+r_{s}}\Delta g_{c}\right)+\frac{1}{f_{c}}\left(\frac{\rho c_{p}}{r_{a}^{2}}\left(T_{c}-T_{\text{atm}}\right)\Delta r_{a}+\rho c_{p}\left(T_{s}-T_{c}\right)\Delta C_{h}\right) & \text{in}\;\Delta T_{c} \end{array}\right.$$

(Equation 15) $$\Delta G=\left\{ \begin{array}{cc} \frac{1}{f_{s}}\left(-\Delta Q_{G}\right)\; & \text{in}\;\Delta T_{s}\\ \frac{1}{f_{s}}\frac{1}{f_{c}}\rho c_{p}C_{h}\left(-\Delta Q_{G}\right) & \text{in}\;\Delta T_{c} \end{array}\right.$$

(Equation 16) $$\Delta AH=\begin{array}{cc} \frac{1}{f_{c}}\left(\Delta Q_{\text{ah}}\right) & \text{in}\;\Delta T_{c} \end{array}$$

#### UHI components

##### Albedo, *α*

The urban surface albedo (*α*_urban_) is influenced by the reflective properties of urban surface materials and the urban geometry (cavity effect).^61^ Considering the radiation exchange process in the urban canopy, the derived *α*_urban_ is more accurate when the reflection is calculated in the urban canopy more times. Numerical simulation studies have shown that it is sufficient to consider only two reflection processes in practice.^62^ The *α*_urban_ was calculated by considering two reflection processes:

(Equation 17) $$\alpha_{\text{urban}}=\frac{2h_{b}\left(\begin{array}{l} \alpha_{w}SVF_{w}+{\alpha_{w}}^{2}SVF_{\text{ww}}SVF_{w}\\ +\alpha_{w}\alpha_{r}SVF_{\text{rw}}SVF_{r} \end{array}\right)+w_{r}\left(\alpha_{r}SVF_{r}+2\alpha_{r}\alpha_{w}SVF_{\text{wr}}SVF_{w}\right)}{2h_{b}+w_{r}}$$

where *h*_b_ is the building height [m], *w*_r_ is the road width [m], *α*_w_ is the albedo of the wall, *α*_r_ is the albedo of the road, and *SVF* denotes the sky view factors calculated as: SVFw=12(hbwr+1−(hbwr)2+1)÷(hbwr)=SVFrw, SVFr=(hbwr)2+1−hbwr, $SVF_{\text{ww}}=1-2SVF_{w}$, $SVF_{\text{wr}}=\frac{1}{2}\left(1-SVF_{r}\right)$.^63^

The rural surface albedo (*α*_rural_) was selected based on the typical surface albedo values provided by the local climate zone (LCZ) system.

The change in albedo induced by urbanization was calculated as follows.

(Equation 18) $$\Delta \alpha =\alpha_{\text{urban}}-\alpha_{\text{rural}}$$

##### Emissivity, ε

Considering the “trapping” effect of urban geometry on long-wave radiation (cavity effect), the effective emissivity of the urban canopy was calculated as^64^:

(Equation 19) $$\varepsilon_{\text{urban}}=\frac{{\varepsilon_{u}}_{,0}}{1-\left(1-{\varepsilon_{u}}_{,0}\right)\left(1-SVF\right)}$$

where *ε*_u,0_ is the emissivity of the urban fabric (assumed to be 0.9^65^) and *SVF* is the sky view factor of the urban canopy, i.e., $SVF={\left\{\cos\left[\arctan\left(\frac{H}{W}\right)\right]\right\}}^{2}$.^66^

Satellite observations show that the emissivity varies little over mostly vegetated surfaces and it deviates only slightly from 0.95 among different land cover types.^57^ Therefore, for vegetation-covered suburban areas, assumed a surface emissivity of: *ε*_rural_ = 0.95.

The change in emissivity induced by urbanization was calculated as follows.

(Equation 20) $$\Delta \varepsilon =\varepsilon_{\text{urban}}-\varepsilon_{\text{rural}}$$

##### Aerodynamic resistance, *r*_a_

The aerodynamic resistance (*r*_a_) was used to represent the degree to which atmospheric turbulence facilitates the transport of heat from a surface. Based on Monin–Obukhov similarity theory, the *r*_a_ values for urban (*r*_a,urban_) and rural (*r*_a,rural_) areas can be modeled as:

(Equation 21) $$r_{a}=\frac{\ln\left(\frac{z_{m}-d}{z_{0,m}}\right)\ln\left(\frac{z_{m}-d}{z_{0,h}}\right)}{{k_{v}}^{2}u\left(z\right)}$$

where *k*_v_ is the von Karman’s constant, *k*_v_ = 0.41, *u*(z) is the wind speed [m·s^–1^], *z*_m_ is the height of wind measurements [m], we assume that the urban effect on wind speed can be neglected at *z*_m_ = 2.5*h*_b_, *u*(z) be assumed equal in urban and rural areas and calculated with the 10m wind speed at the rural weather station, *d* is the zero plane displacement height [m], which was calculated in urban (*d*_u_) and rural (*d*_r_) areas as^67^: $d_{u}=\left[1+{m_{\alpha }}^{-\lambda_{p}}\left(\lambda_{p}-1\right)\right]h_{b}$, $d_{r}=0.67h_{v}$, where *m*_α_ (= 4) is an empirical constant, *λ*_p_ is the plant area density, and *h*_b_ and *h*_v_ are the building and vegetation canopy heights, respectively, i.e., $h_{v}=\frac{22.47}{1+\exp\left[-0.0046\left(P-445.17\right)\right]}$^2^, where *P* is the mean annual precipitation [mm·yr^–1^], *z*_0,m_ and *z*_0,h_ are the roughness lengths for momentum and heat transfer, respectively [m], which were calculated for urban (*z*_0,mu_) and rural (*z*_0,mr_) areas as: $z_{0,\text{mu}}=\left(1-\frac{d_{u}}{h_{b}}\right)\exp\left\{-{\left[0.5\frac{C_{d}}{{k_{v}}^{2}}\left(1-\frac{d_{u}}{h_{b}}\right)\lambda_{f}\right]}^{-0.5}\right\}h_{b}$, $z_{0,\text{mr}}=0.123h_{v}$, where *C*_d_ (= 1.2) is an empirical constant, *λ*_f_ is the frontal area density, i.e., $\lambda_{f}=\frac{VH_{\text{urb}}}{4}$, and *VH*_urb_ is the vertical-to-horizontal urban area ratio; and *z*_0,h_ is usually calculated by $z_{0,h}=0.1z_{0,m}$, although the aerodynamic resistances to heat and momentum transfer are not necessarily monotonically or linearly related, but this simple relationship was adequate for application in this study.^2^

The change in emissivity induced by urbanization was calculated as follows.

(Equation 22) $$\Delta r_{a}=r_{a,\text{urban}}-r_{a,\text{rural}}$$

##### Green cover (*g*_c_) and evapotranspiration

In this study, the effect of difference in urban green cover (*g*_c,u_) on UHIs was considered, where *g*_c,u_ = 0.3 was used as the base case. It was assumed that rural areas are adequately covered by vegetation (*g*_c,r_ = 1). The actual evapotranspiration is controlled by energy and water availability. The water stress factor was used to describe water limitation, $\beta =1+\frac{P}{PET}-{\left[1-I_{r}+{\left(\frac{P}{PET}\right)}^{\omega }\right]}^{1/\omega }$^2^, where *ω* (=2.1) is a model parameter, *I*_r_ (0 ≤ *I*_r_ ≤ 1) represents the level of irrigation, and *PET* is the potential evapotranspiration [mm·yr^–1^], i.e., $PET=\rho \frac{q_{\text{sat}}\left(T_{s}\right)-q_{a}}{r_{a}+r_{s}}\frac{\eta }{\rho_{w}}$, where *ŋ* is a unit conversion factor and *ρ*_w_ is the density of water [kg·m^–3^].

##### Heat storage, *Q*_G_

The OHM was used to parameterize the heat storage (*Q*_G_)^68^:

(Equation 23) $$Q_{G}=\sum_{i=1}^{n}\left(f_{i}a_{1i}\right)Q^{*}+\sum_{i=1}^{n}\left(f_{i}a_{2i}\right)\left(\frac{\partial Q^{*}}{\partial t}\right)+\sum_{i=1}^{n}\left(f_{i}a_{3i}\right)$$

where *i* are different surface types, *f*_*i*_ is the surface fraction occupied by each of the *i* types, and the coefficients a_1_, a_2_, and a_3_ were derived from independent empirical studies,^68^^,^^69^ where a_1_ considers the strength of the mean dependence of *Q*_G_ on the net surface radiation *Q*^∗^ [-], a_2_ considers the degree and direction of the phase relationships between *Q*_G_ and *Q*^∗^ [h], and a_3_ is the intercept term [W·m^–2^].

## Supplemental information

Document S1. Figures S1‒S13 and Tables S1 and S2
