# Supplementary material for: Different explanations for surface and canopy urban heat island effects in relation to background climate
Source: iScience. 2024 Jan 11;27(3):108863. doi: 10.1016/j.isci.2024.108863 (PMC10867416; doi:10.1016/j.isci.2024.108863)
Supplement: Document S1. Figures S1‒S13 and Tables S1 and S2 [file mmc1.pdf]

iScience, Volume *27*

Supplemental information

Different explanations for surface and canopy urban heat island effects in relation to background climate

Liu Yang, Qi Li, Qiong Li, Lei Zhao, Zhiwen Luo, and Yan Liu

Supplementary information

Different explanations for surface and canopy urban heat island effects in relation to background climate

Liu Yang, Qi Li, Qiong Li, Lei Zhao, Zhiwen Luo & Yan Liu

Table S1. The nomenclature in the attribution model

Symbol	Description of the symbol	Symbol	Description of the symbol
C_h	the turbulent transfer coefficient for sensible heat ($s \cdot m^{-1}$)	Q_c	the turbulent exchanges between the air temperature in the urban canyon and atmosphere ($W \cdot m^{-2}$)
COP	the energy efficiency of heat source equipment (-)	Q_E	the latent heat flux ($W \cdot m^{-2}$)
d	the zero plane displacement height (m)	Q_G	the heat storage ($W \cdot m^{-2}$)
d_r	the rural zero plane displacement height (m)	Q_H	the sensible heat flux ($W \cdot m^{-2}$)
d_u	the urban zero plane displacement height (m)	Q_s	the turbulent exchanges between the air temperature in the urban canyon and urban surface ($W \cdot m^{-2}$)
$e_{sat}(T_a)$	the saturation vapor pressure at temperature T_a (kPa)	r_a	the aerodynamic resistance ($s \cdot m^{-1}$)
E_{out}	the latent heat pumped out from the building ($W \cdot m^{-2}$)	$r_{a,rural}$	the rural aerodynamic resistance ($s \cdot m^{-1}$)
g_c	the green cover (-)	$r_{a,urban}$	the urban aerodynamic resistance ($s \cdot m^{-1}$)
$g_{c,r}$	rural green cover (-)	r_s	the surface resistance ($s \cdot m^{-1}$)
$g_{c,u}$	urban green cover (-)	S^*	the net short-wave radiation ($W \cdot m^{-2}$)
h	the convective heat transfer coefficient ($W \cdot m^{-2} \cdot K^{-1}$)	S_{in}	the incoming short-wave radiation ($W \cdot m^{-2}$)
h_b	the building height (m)	SVF	the sky view factor (-)
h_v	the vegetation canopy height (m)	T_a	the air temperature (K)
H_{out}	the sensible heat pumped out from the building ($W \cdot m^{-2}$)	T_{atm}	the temperature of the atmosphere above the urban canopy (K)
$I_{r,u}$	urban irrigation index (-)	T_c	the canopy air temperature (K)
k_v	the von Karman's constant (-)	T_{in}	the indoor air temperature (K)
l_v	the latent heat of vaporization ($J \cdot kg^{-1}$)	T_s	the surface temperature (K)
L^*	the net long-wave radiation ($W \cdot m^{-2}$)	$u(z)$	the wind speed ($m \cdot s^{-1}$)
L_{in}	the incoming long-wave radiation ($W \cdot m^{-2}$)	v_a	the total ventilation rate in the building ($m^3 \cdot h^{-1}$)
L_{out}	the upward long-wave radiation ($W \cdot m^{-2}$)	VH_{urb}	the vertical-to-horizontal urban area ratio (-)
p_{atm}	the atmospheric pressure (kPa)	w_b	the building width (m)
P	mean annual precipitation ($mm \cdot yr^{-1}$)	w_r	the road width [m]
PET	potential evapotranspiration ($mm \cdot yr^{-1}$)	z_m	the height of wind measurements (m)
q_a	the specific humidity of air ($kg \cdot kg^{-1}$)	$z_{0,h}$	the roughness lengths for heat transfer (m)
q_{in}	the specific humidity of the indoor air ($kg \cdot kg^{-1}$)	$z_{0,m}$	the roughness lengths for momentum (m)
$q_{sat}(T_s)$	the saturated specific humidity at temperature T_s ($kg \cdot kg^{-1}$)	$z_{0,mr}$	the rural roughness lengths for momentum (m)
Q^*	the net surface radiation ($W \cdot m^{-2}$)	$z_{0,mu}$	the urban roughness lengths for momentum (m)
Q_{ah}	the anthropogenic heat flux ($W \cdot m^{-2}$)		

Table S2. The greek letter and abbreviation in the attribution model

Symbol	Description of the symbol	Symbol	Description of the symbol
		Greek letters	
c_p	the specific heat of air at constant pressure ($J\cdot kg^{-1}\cdot K^{-1}$)	λ_f	the frontal area density (-)
α	surface albedo (-)	λ_p	the plant area density (-)
α_r	the albedo of the road (-)	σ	the Stefan–Boltzmann constant ($W\cdot m^{-2}\cdot K^{-4}$)
α_{rural}	the rural surface albedo (-)	β	the water stress factor (-)
α_{urban}	the urban surface albedo (-)	β_{in}	the thermal efficiency of the total heat exchanger (-)
α_w	the albedo of the wall (-)	φ_p	the ratio of hourly occupants relative to the peak number of occupants per floor area (-)
ρ	the air density ($kg\cdot m^{-3}$)	ΔAH	the urban–rural differences in the anthropogenic heat ($W\cdot m^{-2}$)
ρ_b	building density (-)	ΔCV	the urban–rural differences in the convection efficiency ($W\cdot m^{-2}$)
ρ_w	the density of water ($kg\cdot m^{-3}$)	ΔET	the urban–rural differences in the evapotranspiration ($W\cdot m^{-2}$)
ε_a	atmospheric emissivity (-)	ΔG	the urban–rural differences in the heat storage ($W\cdot m^{-2}$)
ε_{rural}	the rural surface emissivity (-)	ΔT	the urban–rural differences in the temperature (°C)
ε_s	the surface emissivity (-)	ΔT_c	the canopy urban heat island intensity (°C)
$\varepsilon_{u,0}$	the emissivity of the urban fabric (-)	ΔT_s	the surface urban heat island intensity (°C)
ε_{urban}	the urban surface emissivity (-)	ΔR^*	the urban–rural differences in the net radiation ($W\cdot m^{-2}$)
		Abbreviations	
AH	anthropogenic heat	LST	land surface temperature
CUHI	canopy urban heat island	SD	standard deviation
CV	convection efficiency	SUHI	surface urban heat island
ET	evapotranspiration	R^*	net radiation
G	heat storage	UHI	urban heat island

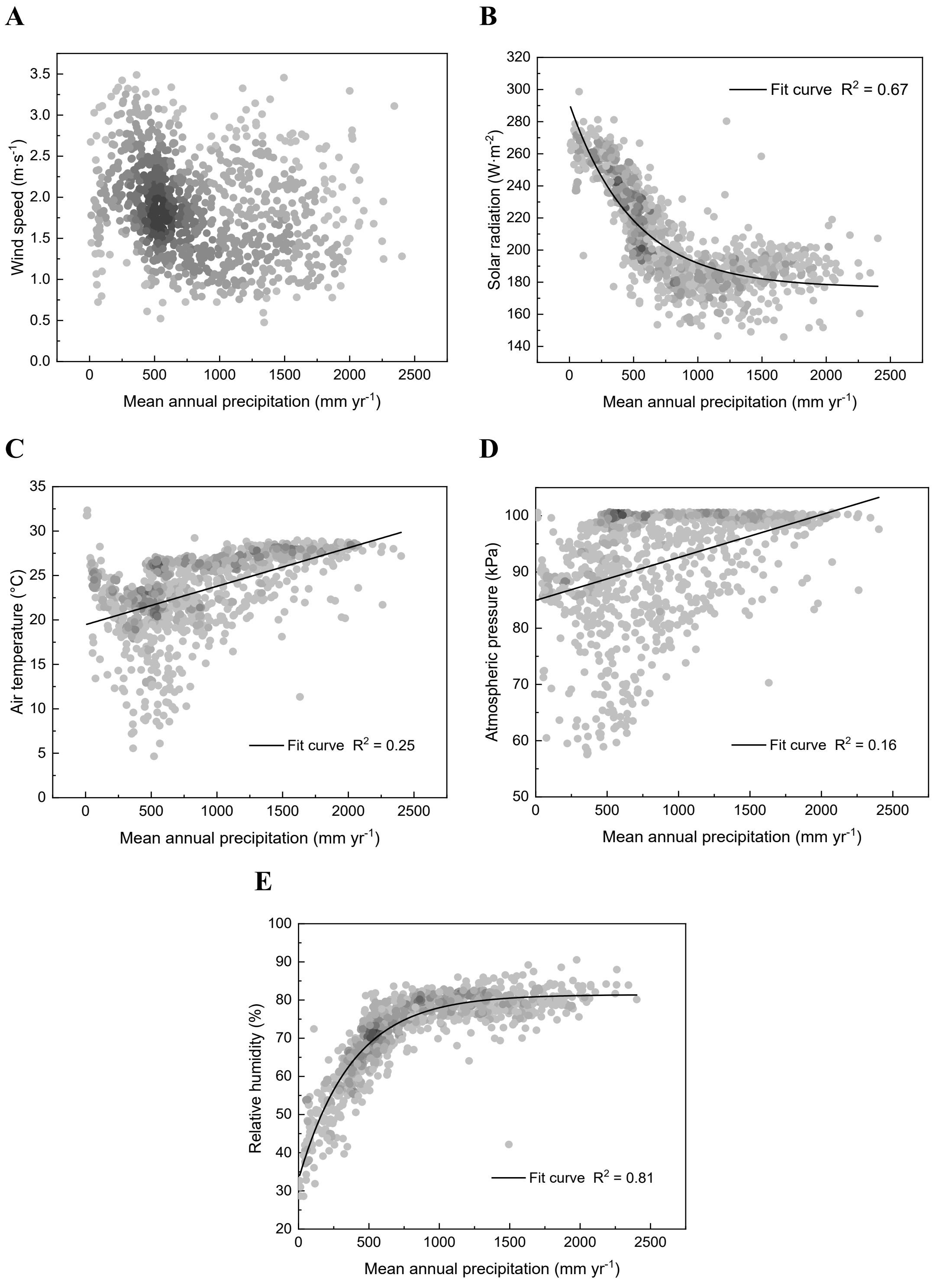

Figure S1. Relationships between urban background climate conditions and precipitation.

(A) wind speed, (B) solar radiation, (C) air temperature, (D) atmospheric pressure, and (E) relative humidity.

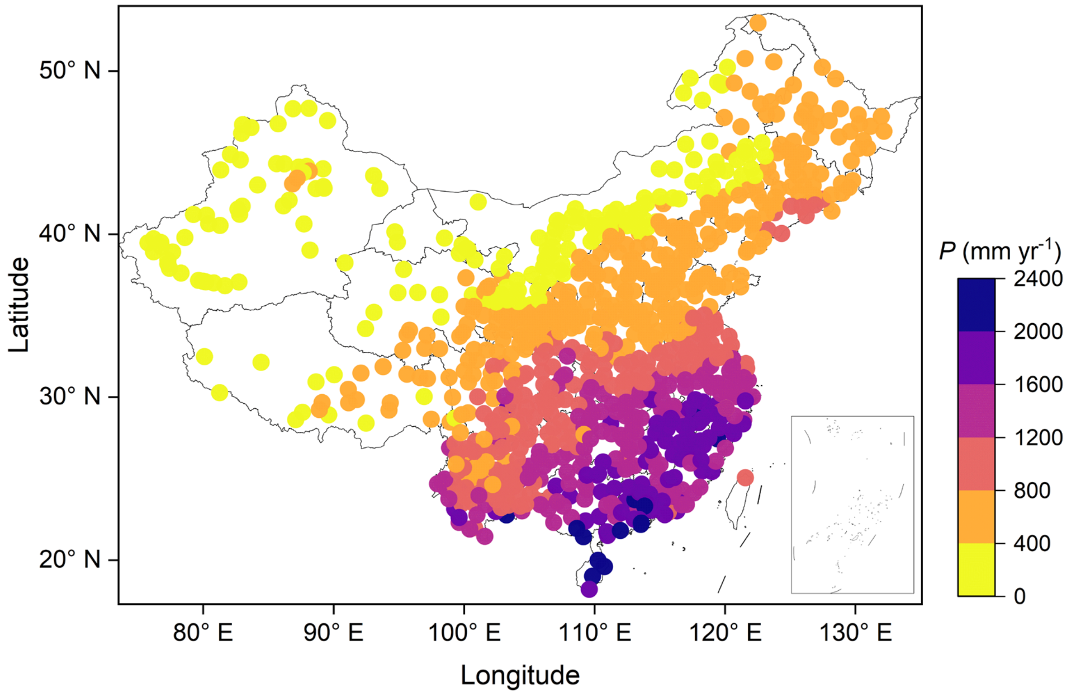

Figure S2. Distribution of mean annual precipitation (*P*) in China.

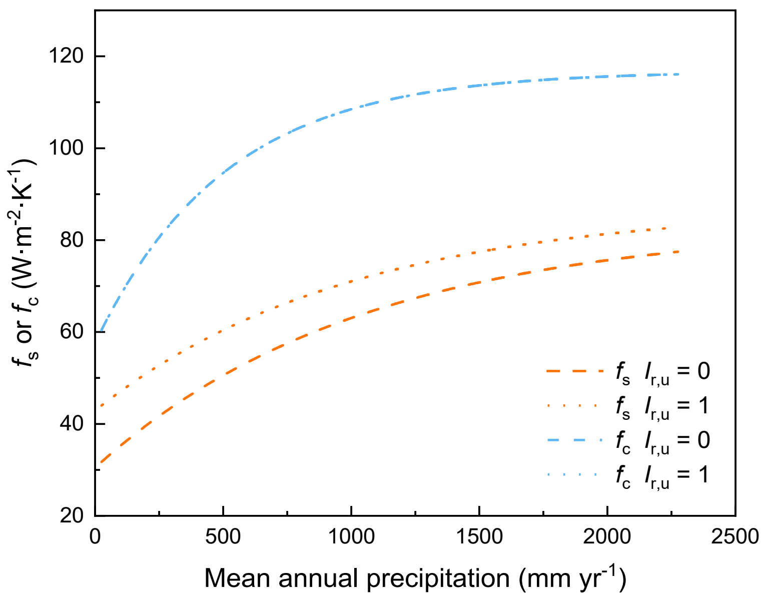

Figure S3. Effects of precipitation on energy redistribution factors f_s and f_c.

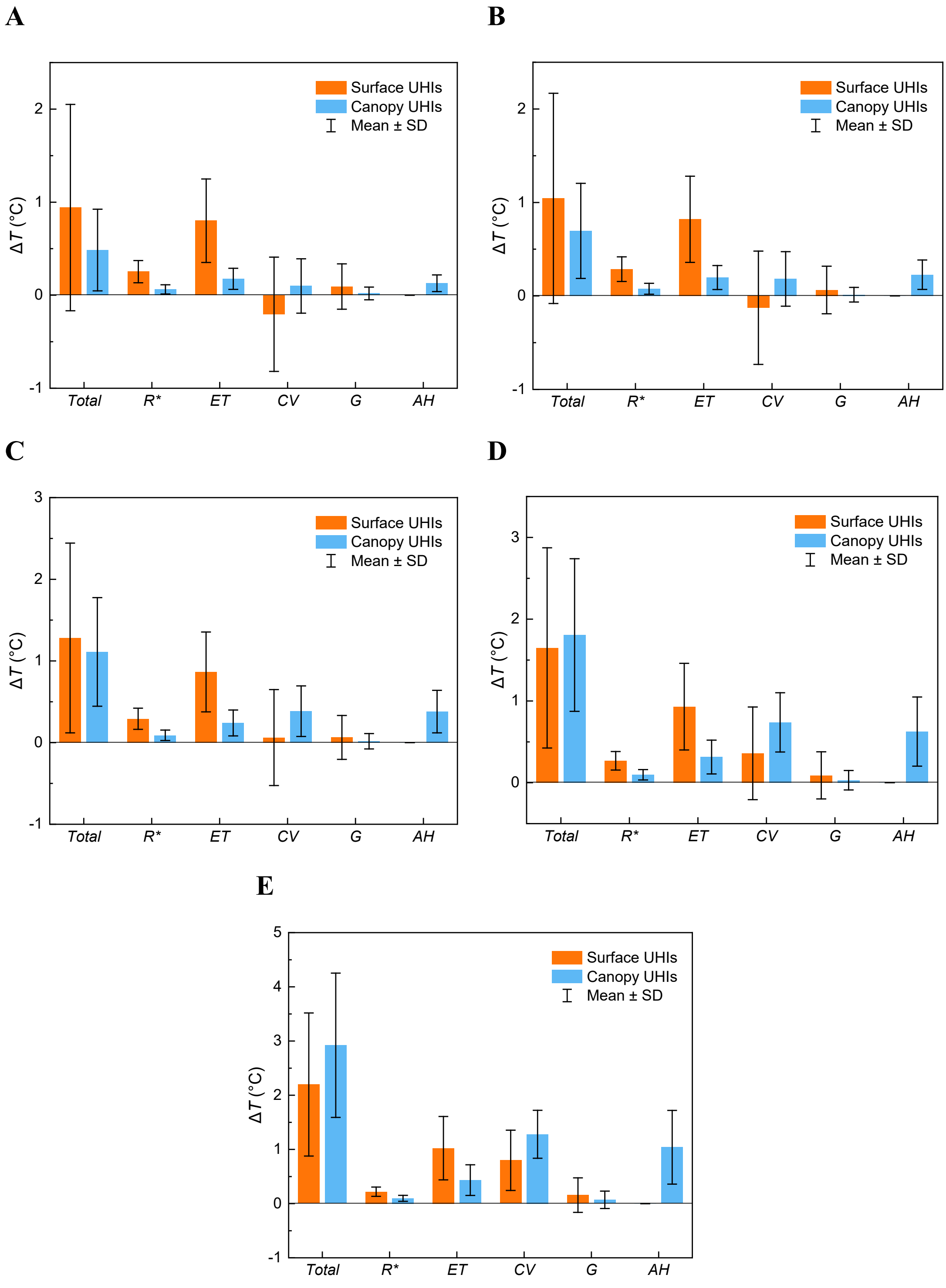

Figure S4. Attribution of summer surface and canopy urban heat islands (UHIs) under different building densities.

Summer UHIs (surface UHIs, orange; canopy UHIs, blue) and their components at: (A) $\rho_b = 0.2$, (B) $\rho_b = 0.3$, (C) $\rho_b = 0.4$, (D) $\rho_b = 0.5$ and (E) $\rho_b = 0.6$. Error bars indicate ± 1 standard deviation.

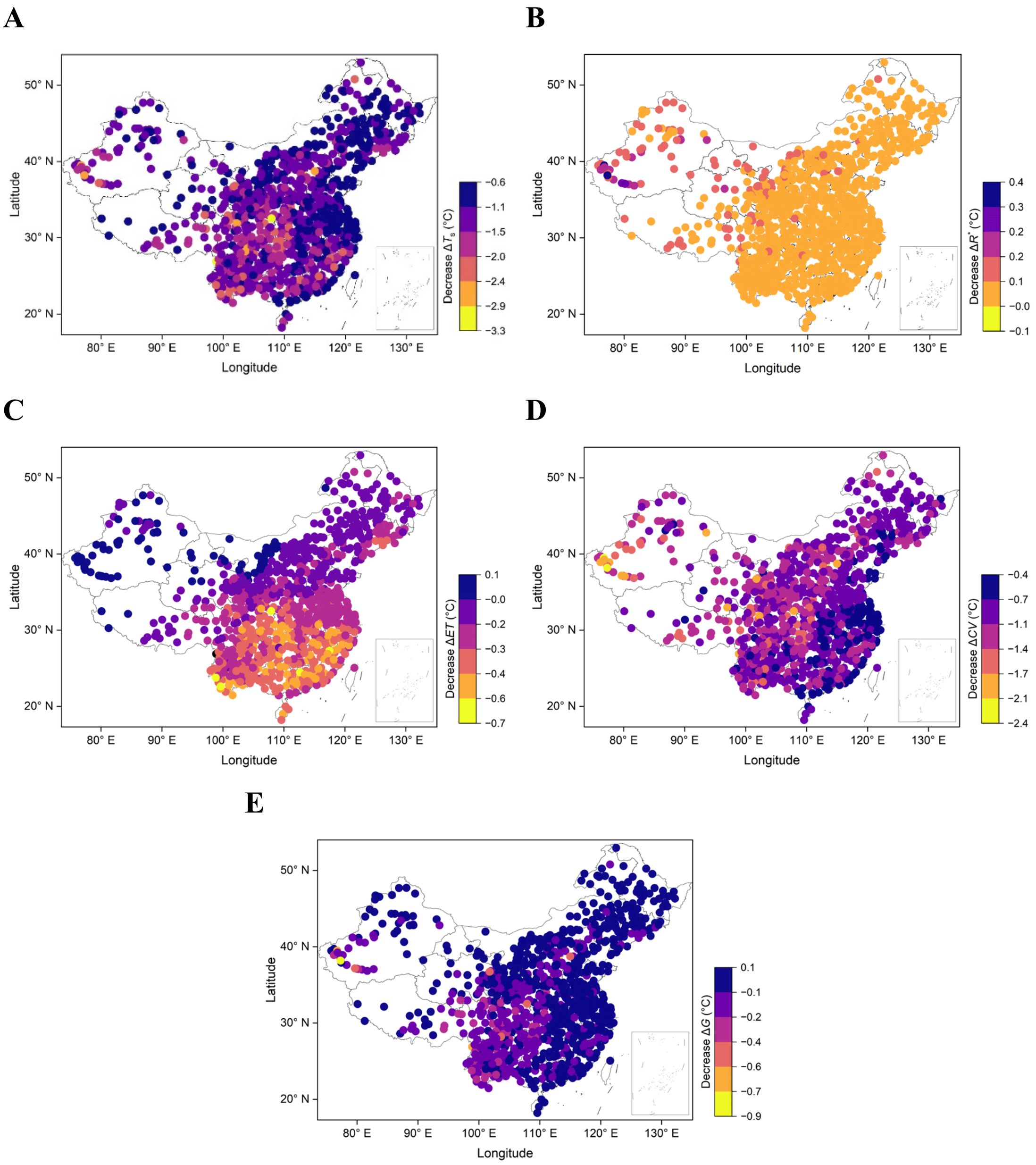

Figure S5. Impact of urban morphology on the intensity of surface urban heat island mitigation by reducing the building density.

Distribution of surface urban heat island effect and its components mitigation by reducing the building density ($\rho_b = 0.6$ to $\rho_b = 0.2$): (A-E) are ΔT_s, ΔR^*, ΔET, ΔCV and ΔG, respectively.

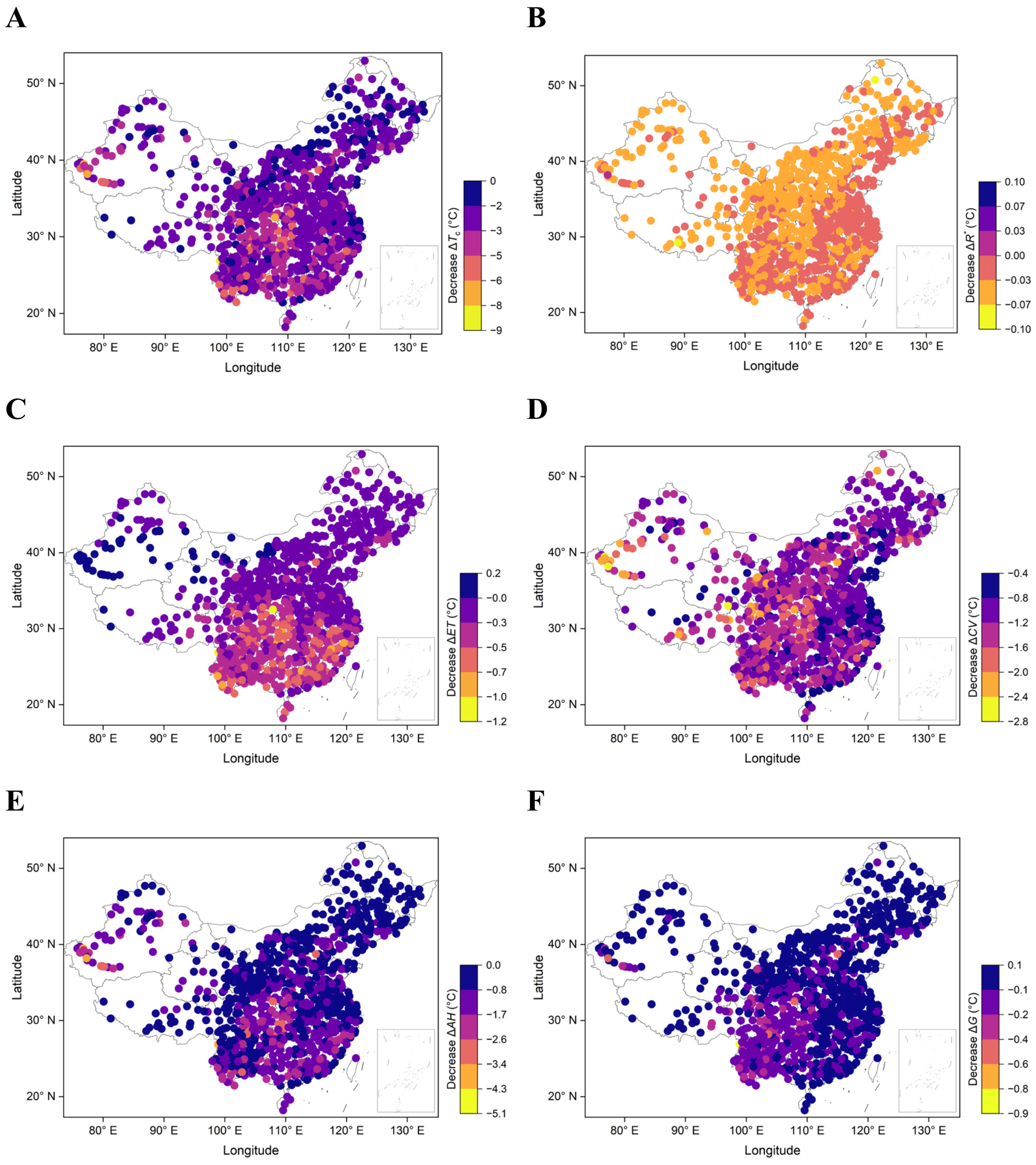

Figure S6. Impact of urban morphology on the intensity of canopy urban heat island mitigation by reducing the building density.

Distribution of canopy urban heat island effect and its components mitigation by reducing the building density ($\rho_b = 0.6$ to $\rho_b = 0.2$): (A-F) are ΔT_c, ΔR^*, ΔET, ΔCV, ΔAH and ΔG, respectively.

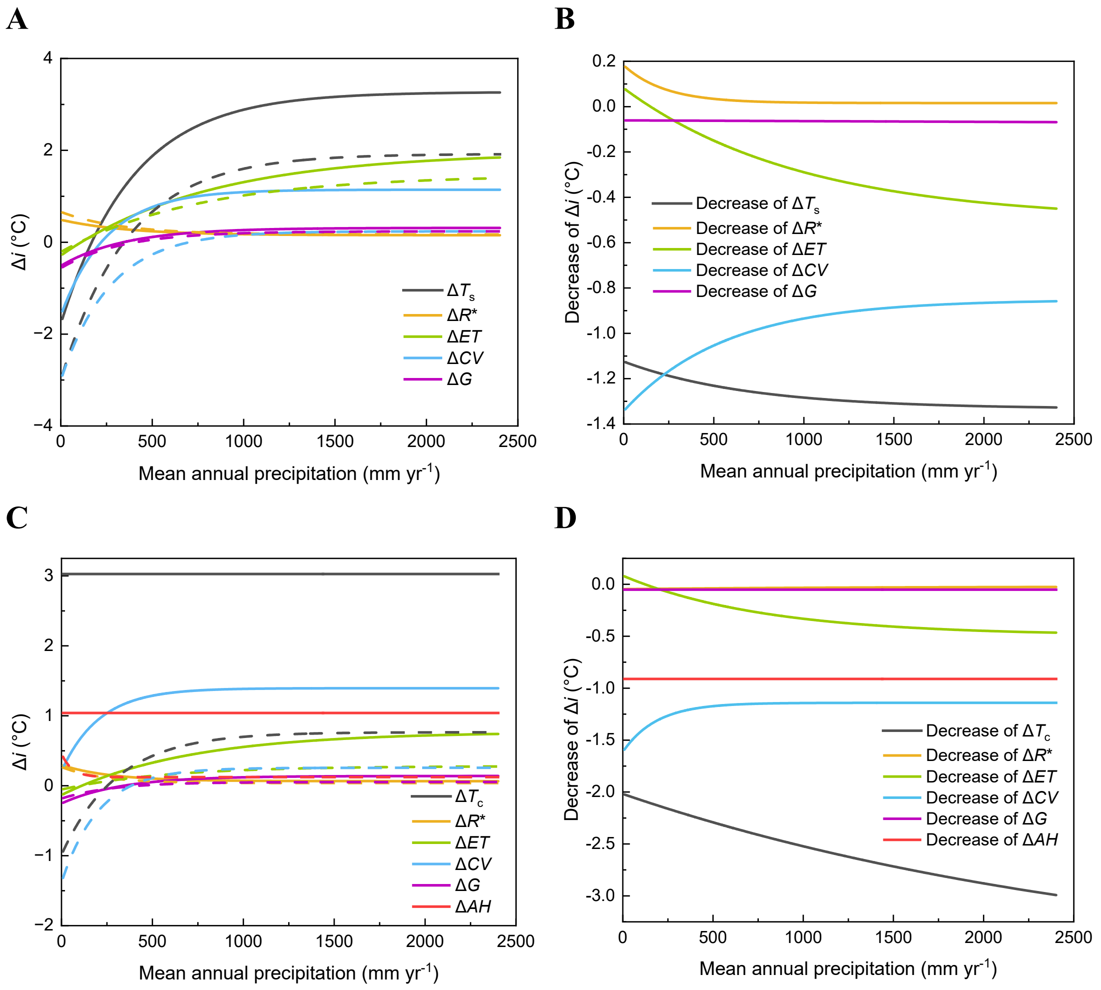

Figure S7. Effects of precipitation on the intensity of urban heat island at different building density (ρ_b = 0.6 and ρ_b = 0.2).

Summer urban heat islands and components at ρ_b = 0.6 (solid lines) and ρ_b = 0.2 (dash lines): (A) ΔT_s, (C) ΔT_c. Summer urban heat islands mitigation by reducing the building density (ρ_b = 0.6 to ρ_b = 0.2): (B) ΔT_s, (D) ΔT_c.

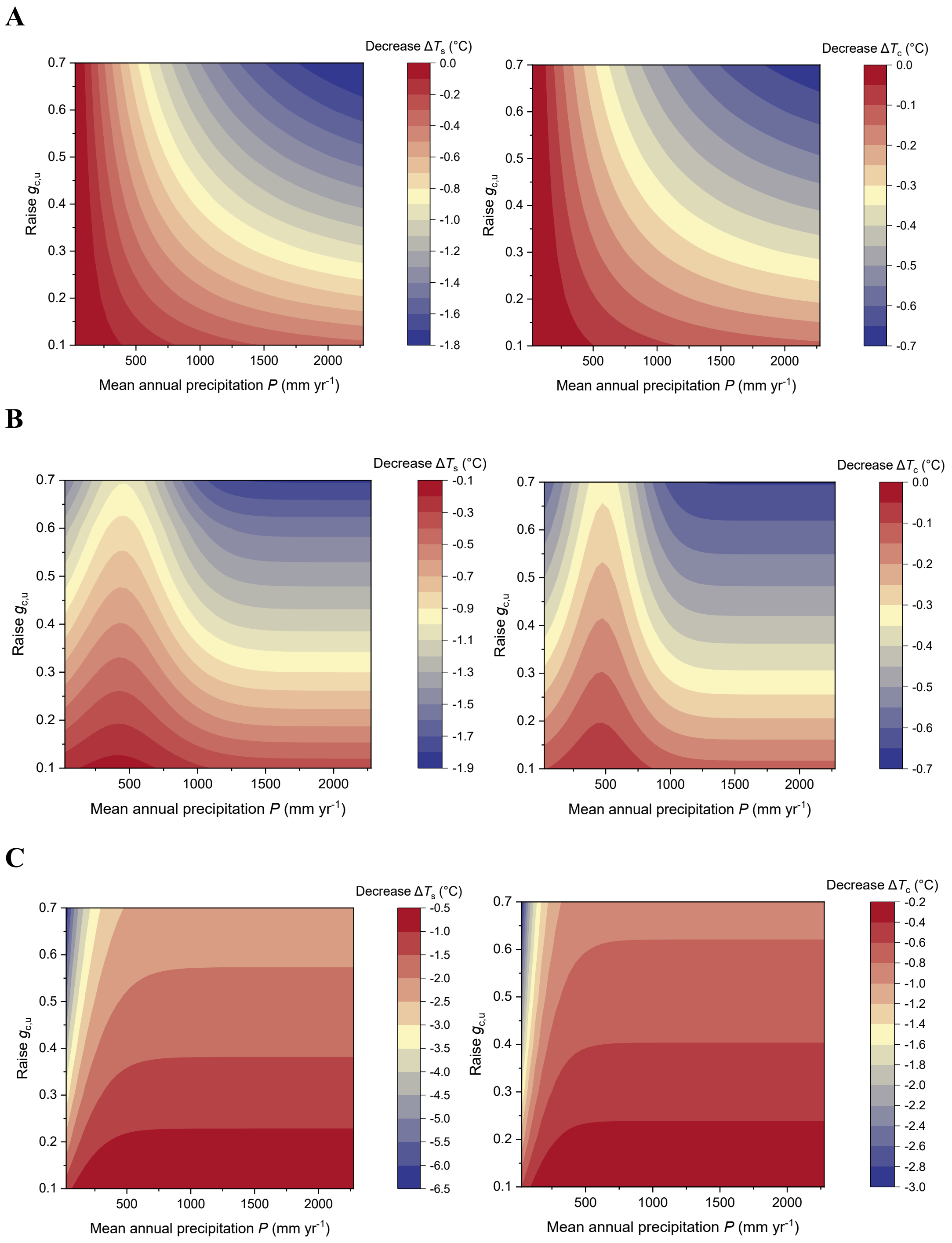

Figure S8. Impact of urban background climate on the intensity of urban heat island mitigation by increasing the urban green cover.

Urban green cover of 0 was set as a base case and the impacts of different urban irrigation indexes were considered: (A) $I_{r,u} = 0$, (B) $I_{r,u} = 0.3$, and (C) $I_{r,u} = 1$.

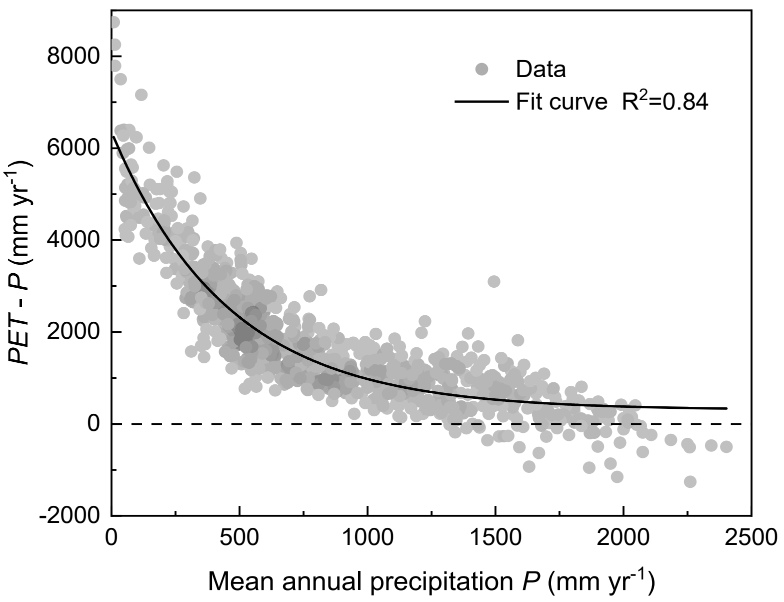

Figure S9. Impact of urban background climate on the difference between potential evapotranspiration (*PET*) and mean annual precipitation (*P*) (*PET* – *P*).

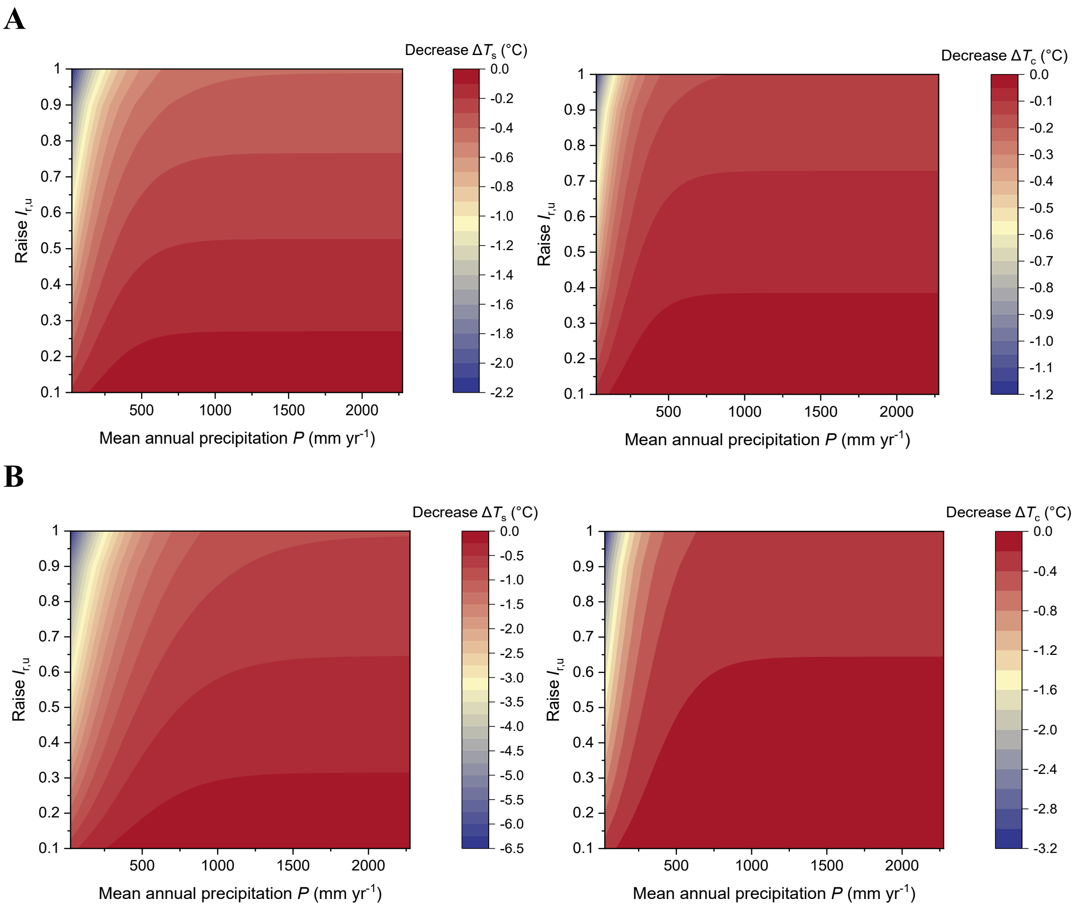

Figure S10. Impact of urban background climate on the intensity of urban heat island mitigation by increasing the urban irrigation index.

The urban irrigation index was set to 0 as a base case and the impacts of different amounts of urban green cover were determined: (A) $g_{c,u} = 0.15$ and (B) $g_{c,u} = 0.7$.

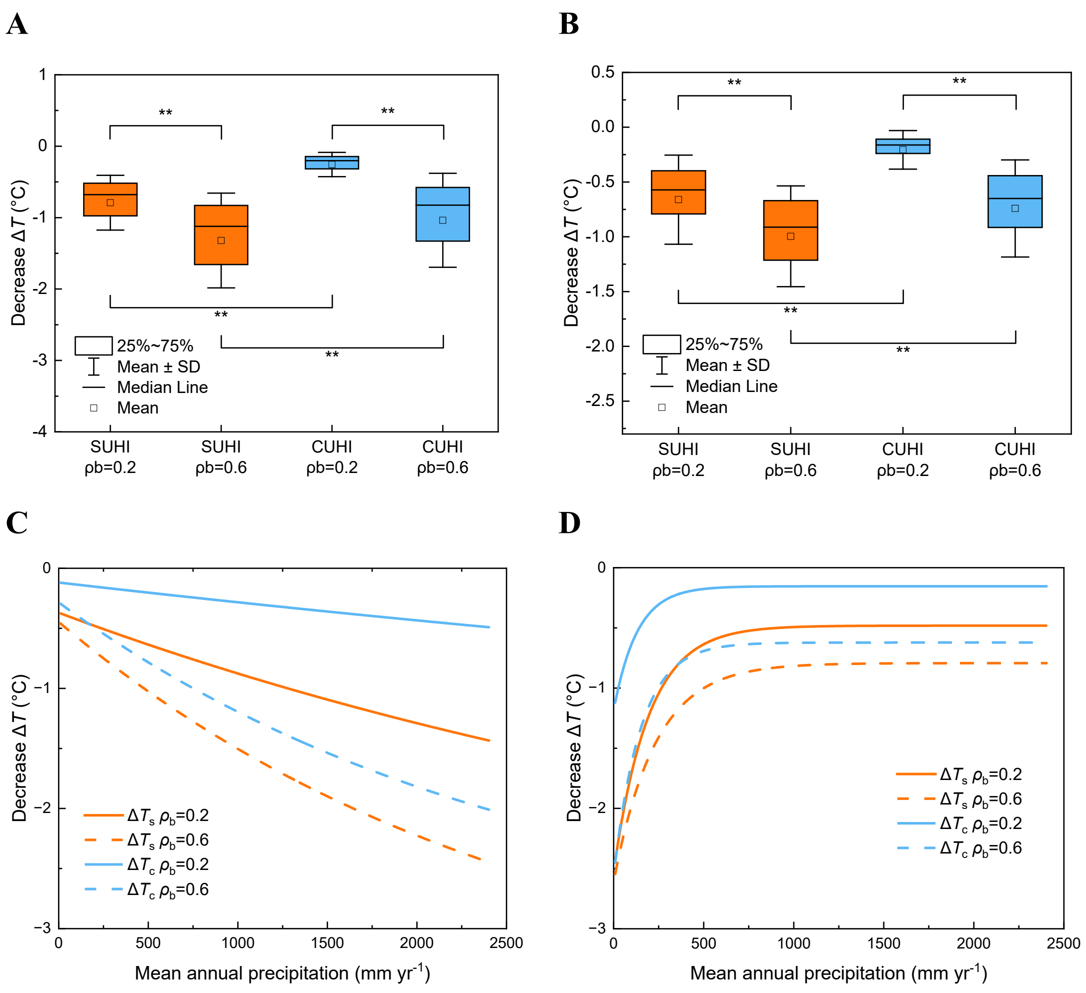

Figure S 11. Impact of urban morphology on the effectiveness of heat mitigation strategies.

Significance analysis of the effectiveness of heat mitigation strategies: (A) increasing the urban green cover, from $g_{c,u}$ = 0 to $g_{c,u}$ = 0.4, (B) increasing the irrigation index, from $I_{r,u}$ = 0 to $I_{r,u}$ = 1. ** denote significant differences at P = 0.01 level. Effects of precipitation on the intensity of urban heat island mitigation by heat mitigation strategies at different building density: (C) increasing the urban green cover, from $g_{c,u}$ = 0 to $g_{c,u}$ = 0.4, (D) increasing the irrigation index, from $I_{r,u}$ = 0 to $I_{r,u}$ = 1. Error bars indicate ± 1 standard deviation.

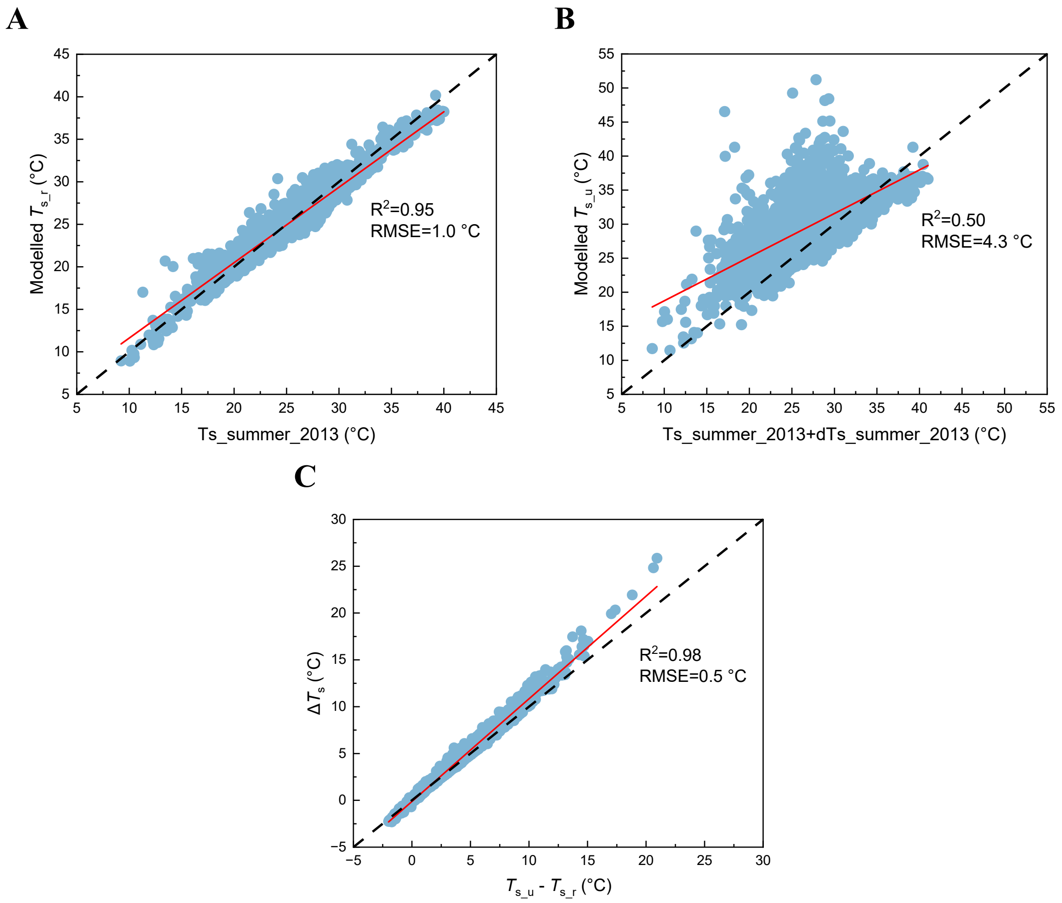

Figure S12. Surface energy balance model validation.

Model validation is performed with the 2013 Global Urban Heat Island Dataset, the background climate data are retrieved from the Modern Era Retrospective-Analysis for Research and Applications (MERRA), urban morphology is calculated according to the Urban relations provided by Manoli et al [2]. We selected cities with populations over 10^5 for model validation. (A-B) Comparison of the surface temperature observed and simulated by Eq. 4, (A) in rural area and (B) in urban area, the observed urban surface temperature was obtained from the observed rural surface temperature + urban heat island intensity (i.e. Ts_summer_2013 + dTs_summer_2013). (C) The error caused by the first-order Taylor series expansion, comparison of the simulated by mechanistic attribution model (Eq .5) and the difference between simulated urban surface temperature and rural surface temperature (Eq. 4).

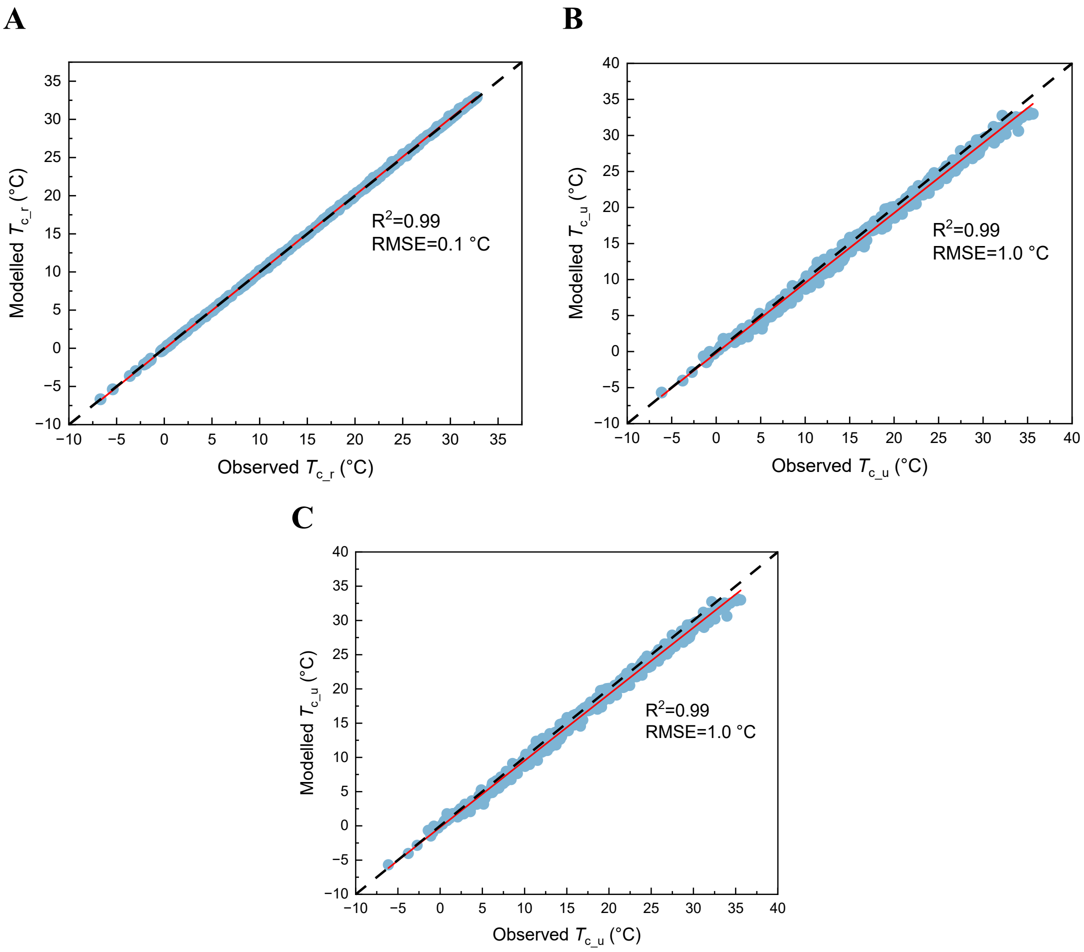

Figure S13. Canopy energy balance model validation.

Model validation is performed with the observed data in Xi'an, China, in 2016, which available from China building energy efficiency design basic data platform (https://buildingdata.xauat.edu.cn/). The hourly meteorological data are averaged to obtain daily data for the model inputs. The urban morphology is set to: h_b = 15 m, ρ_b = 0.3 and w_b = 15 m. (A-B) Comparison of the canopy air temperature observed and simulated by Eq .8, (A) in rural area and (B) in urban area. (C) The error caused by the first-order Taylor series expansion, comparison of the simulated by mechanistic attribution model (Eq .9) and the difference between simulated urban s canopy air temperature and rural canopy air temperature (Eq .8).
